# Impact of endoplasmic reticulum aminopeptidases 1 (ERAP1) and 2 (ERAP2) on neutrophil cellular functions

**DOI:** 10.3389/fcell.2024.1506216

**Published:** 2025-01-07

**Authors:** Irma Saulle, Fiona Limanaqi, Micaela Garziano, Maria Luisa Murno, Valentina Artusa, Sergio Strizzi, Matteo Giovarelli, Carsten Schulte, Jacopo Aiello, Mario Clerici, Claudia Vanetti, Mara Biasin

**Affiliations:** ^1^ Dipartimento di Scienze Biomediche e Cliniche, Università degli Studi di Milano, Milano, Italy; ^2^ Dipartimento di Fisiopatologia Medico-Chirurgica e dei Trapianti, Università degli Studi di Milano, Milano, Italy; ^3^ Istituti di Ricovero e Cura a Carattere Scientifico (IRCCS), Fondazione Don Carlo Gnocchi, Milano, Italy

**Keywords:** ERAP1, ERAP2, neutrophils, cell migration, phagocytosis, autophagy

## Abstract

**Introduction:**

Endoplasmic reticulum aminopeptidases 1 (ERAP1) and 2 (ERAP2) modulate a plethora of physiological processes for the maintenance of homeostasis in different cellular subsets at both intra and extracellular level.

**Materials and methods:**

In this frame, the extracellular supplementation of recombinant human (rh) ERAP1 and ERAP2 (300 ng/ml) was used to mimic the effect of stressor-induced secretion of ERAPs on neutrophils isolated from 5 healthy subjects. In these cells following 3 h or 24 h rhERAP stimulation by Western Blot, RT-qPCR, Elisa, Confocal microscopy, transwell migration assay, Oxygraphy and Flow Cytometry we assessed: i) rhERAP internalization; ii) activation; iii) migration; iv) oxygen consumption rate; v) reactive oxygen species (ROS) accumulation; granule release; vi) phagocytosis; and vii) autophagy.

**Results:**

We observed that following stimulation rhERAPs: i) were internalized by neutrophils; ii) triggered their activation as witnessed by increased percentage of MAC-1^+^CD66b^+^ expressing neutrophils, cytokine expression/release (IL-1β, IL-8, CCL2, TNFα, IFNγ, MIP-1β) and granule enzyme secretion (myeloperoxidase, Elastase); iii) increased neutrophil migration capacity; iv) increased autophagy and phagocytosis activity; v) reduced ROS accumulation and did not influence oxygen consumption rate.

**Conclusion:**

Our study provides novel insights into the biological role of ERAPs, and indicates that extracellular ERAPs, contribute to shaping neutrophil homeostasis by promoting survival and tolerance in response to stress-related inflammation. This information could contribute to a better understanding of the biological bases governing immune responses, and to designing ERAP-based therapeutic protocols to control neutrophil-associated human diseases.

## Introduction

The endoplasmic reticulum aminopeptidase 1 (ERAP1) and its paralog 2 (ERAP2), are members of the zinc metalloprotease family of aminopeptidases that are ubiquitously expressed in human cells (Tsujimoto and Hattori, 2005; Agrawal and Brown, 2014). Their main function in antigen processing and presentation, as homo or heterodimers, hinges on the meticulous trimming of peptides to a length suitable to fit into the groove of a major histocompatibility complex (MHC) class I molecule (Kloetzel and Ossendorp, 2004; Mattorre et al., 2022). The expression of peptide-MHC-I complexes on the cellular membrane, in turn, orchestrates a delicate balance ensuring optimal presentation to antigen-specific CD8^+^ T cells (Chen et al., 2016; Li et al., 2019; Tedeschi et al., 2023), thus promoting the defence against viruses (Saulle et al., 2020a; Saulle et al., 2020b), malignancies (Dersh et al., 2021; Dhatchinamoorthy et al., 2021), and other endogenous antigens (Goldberg and Bona, 2011; Dawson and Levings, 2017).

Recent evidence suggest a broader role for ERAP1 and ERAP2 in the regulation of a number of physiological processes relevant for the maintenance of homeostasis in different cellular subsets (Tsujimoto and Hattori, 2005; Evnouchidou et al., 2023; Limanaqi et al., 2023), challenging the conventional view of these enzymes solely as guardians of antigen presentation. For instance, ERAP1 has been shown to work as a critical controller of inflammasome-mediated proinflammatory and ER stress responses (Blake et al., 2022a), while ERAP2 has been recently demonstrated to induce unfolded protein response (UPR)-mediated autophagy in pancreatic stellate cells (PSCs) (Guan et al., 2022). Unexpectedly, a number of recent evidence also indicate that following inflammatory stimuli ERAP molecules may be secreted both as soluble proteins or cargo inside extracellular vesicle (EV) (Goto et al., 2014; 2015; 2018; Saulle et al., 2019). Once in the extracellular milieu, ERAPs may govern additional functions which have been only partially elucidated. These include: 1) phagocytosis enhancement (Goto et al., 2014; Saulle et al., 2019), inflammasome assembly (Saulle et al., 2019; Blake et al., 2022a) and NO synthesis from monocyte/macrophage lineage (Goto et al., 2014; 2015); 2) shedding of the extracellular portion of cytokine receptors (Cui et al., 2002; Cui et al., 2003a; Cui et al., 2003b); 3) Vascular Endothelial Growth Factor (VEGF)-dependent neo-angiogenesis regulation (Yamazaki et al., 2004); 4) renin angiotensin system (RAS) modulation (D’Amico et al., 2021; Mattorre et al., 2022).

Given the multitude of biological functions that can be controlled by ERAPs, it is not surprising that their expression has been associated with susceptibility/progression/control of a myriad of diseases (Reeves et al., 2020). Indeed, increased ERAP1 and ERAP2 expression was demonstrated to raise the odds of developing a number of autoimmune diseases such as Chron, Beçhet’s disease, Birdshot Chorioretinopathy and Type I diabetes (Alvarez-Navarro et al., 2015; Castro-Santos et al., 2017; Guasp et al., 2019; Limanaqi et al., 2023). Conversely, in tumours, upregulation of ERAP1 and ERAP2 expression has been generally associated with an increase in protective immune cell infiltration and a higher efficacy of immune checkpoint inhibitor therapy (Yang et al., 2021). As for viral infections, the scientific community agrees upon a protective role exerted by ERAPs in controlling most viral infections and disease progression, although available data are still limited (Biasin et al., 2013; Liu et al., 2017; Saulle et al., 2020a; 2020b; Klunk et al., 2022).

**FIGURE 1:**
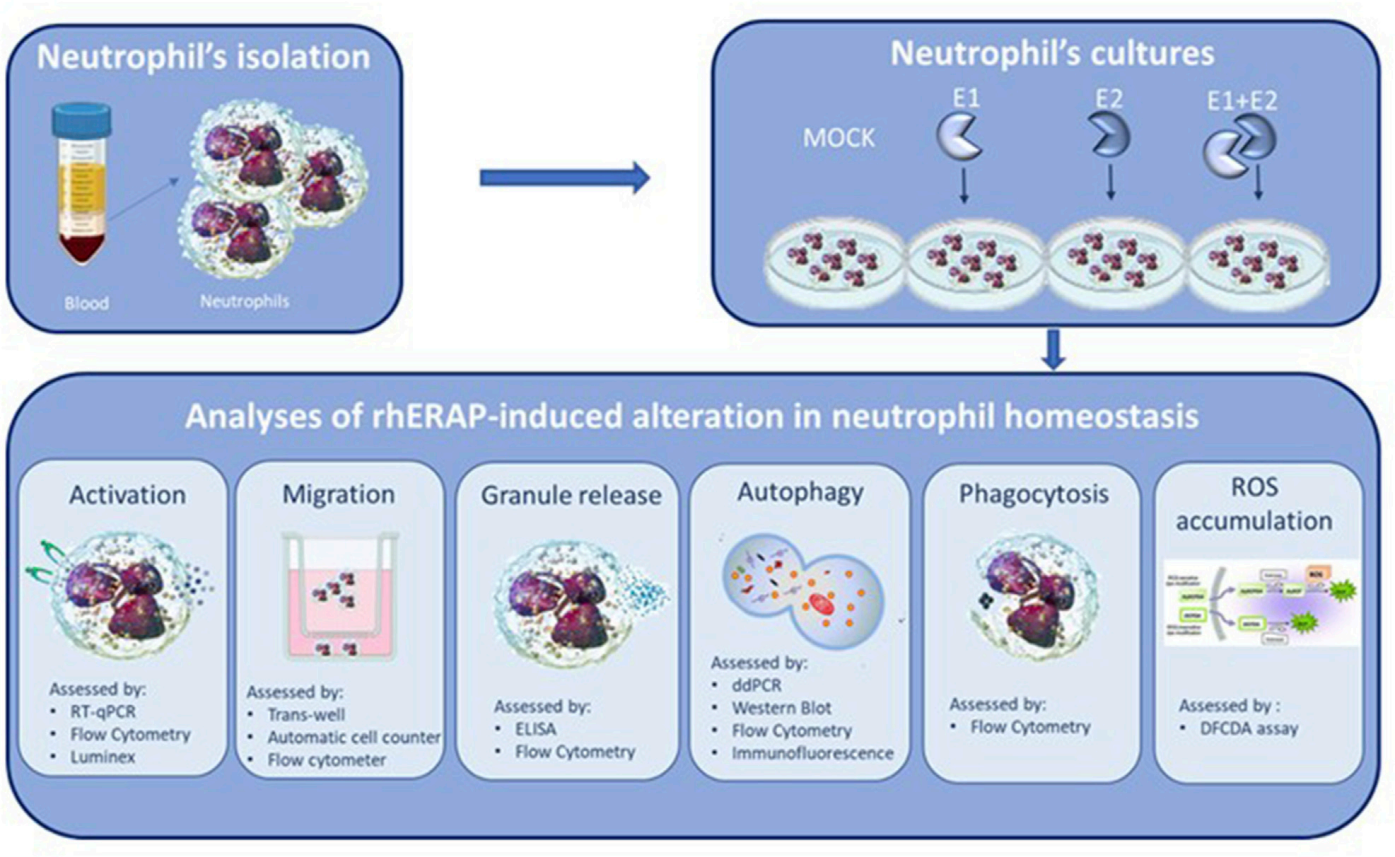
Synoptic representation of the study design.

Based on these premises, in this study, the extracellular supplementation of recombinant human (rh) ERAP1 and ERAP2 was used to mirror the effect of their stressor-induced secretion on a less-explored facet of innate immunity: the neutrophils. Neutrophils are the most abundant leukocytes in the natural immune system (Rosales, 2018) and are largely involved in the modulation of all the above-mentioned ERAP-associated human diseases (Segal, 2018; Bousquet et al., 2022; Yan et al., 2022). Indeed, they are the first cellular population to be recruited during infections (Galani and Andreakos, 2015), play crucial roles in the onset and progression of autoimmune diseases (Bissenova et al., 2022), and are emerging as important regulators of tumour growth (Hedrick and Malanchi, 2022). In the attempt to obtain an exhaustive overview of the cellular pathways potentially affected by soluble ERAPs, in neutrophils isolated from healthy subjects, we investigated downstream biological functions upon addition of rhERAP1 (E1) and rhERAP2 (E2). In particular, we focused on activation, migration, reactive oxygen species (ROS) release, secretion of cytolytic enzymes/cytokines, phagocytosis and autophagy (Figure 1).

Gathering novel information on ERAP-induced mechanisms of action and cellular targets in neutrophils could help both in a better understanding of the biological bases governing immune responses and the design of ERAP-based pharmacological approaches in the context of specific diseases.

## Materials and methods

### ERAP1 and ERAP recombinant human (rh) proteins

Recombinant human ERAP1 (E1) and ERAP2 (E2) were purchased from R&D (ERAP1-Catalog Number: 2334-ZN; ERAP2- Catalog Number: 3830-ZN) (R&D system, Minneapolis, United States). Both, ERAP1 and ERAP2 were expressed in a mouse myeloma cell line (NS0) and display enzymatic activity. The lyophilized protein (10 μg) was reconstituted in a 0.2 μm filtered solution in Tris, NaCl and Glycerol at a final concentration of 300 ng/μL 5 µL aliquots were prepared and stored at −20°C. The enzymatic activity was measured by their ability to cleave the fluorogenic peptide substrate, Arg¬7¬amido-4¬methylcoumarin (Arg¬AMC) as suggested by the datasheet. Results were analysed by Varioskan (Thermofisher) at excitation and emission wavelengths of 380 nm and 460 nm (top read), respectively, in kinetic mode for 5 min.

A limulus amebocyte lysate (LAL) chromogenic kit (# HIT302, Hycult Biotech) with a minimum detection limit of 0.04 EU/mL was used to measure endotoxin levels before treatment. Endotoxin levels were <0.1 EU/mL and the proteins were used at a final concentration of 300 ng/mL based on previous (Aldhamen et al., 2015; Saulle et al., 2021; Niu et al., 2022) and our pilot tests.

### Neutrophil isolation and cell culture conditions

Neutrophils were obtained from the venous blood of 5 healthy volunteers. Ethical clearance was obtained from the University of Milan Ethics Committee (number 14/22). Written informed consent was obtained after receiving information about the use of their biological samples. The biological material was anonymized.

Neutrophil preparations were isolated by Ficoll-Hypaque density centrifugation followed by dextran sedimentation for 20 min (Stie and Jesaitis, 2004). After the incubation with sedimental liquid (3% Gelatin, 0.9% NaCl and 0.1% Dextrose) neutrophil containing supernatant was collect, centrifuged at 1,800 rpm for 5 min at RT and red blood cells were lysed with ammonium-potassium chloride (ACK) lysis buffer (Euroclone, Milan, Italy). Neutrophils were then washed, centrifuged and the pellet were resuspended in RPMI 1640 medium (Euroclone, Milan, Italy) (supplemented with 2% L-Glutamine 100 × 200 mM and 1% Penicillin-Streptomycin Solution 100X) with 10% FBS (*fetal bovine serum*) (Euroclone, Milan, Italy). Experiments were performed when cell purity was >95% as verified by flow cytometry, assessing to the following markers: CD15^+^ (Kromo Orange Bio-Legend) CD16^++^(Pacific Blue Beckman Coulter) CD45^dim^ (ECD Bio-Legend). When >5% of contaminating eosinophils were detected in some preparations, the samples were excluded from the protocol. Since the average half-life of circulating neutrophils, in physiologic condition is about 1 day, all our experiments were performed over 24 h (Pillay et al., 2010).

Neutrophils for all experiment were treated with or without 300 ng/mL of E1 or 300 ng/mL E2 or 150 ng/mL E1 + 150 ng/mL E2 (Bio-Techne McKinley Place NE Minneapolis, United States).

For gene expression analysis and migration assay neutrophils were treated with rhERAPs for 3 h while for flow cytometry, autophagy and western blot analysis the neutrophils were challenged with rhERAPs for 24 h. Results were always compared with the untreated condition (CTR). All the experiments were performed in duplicate for each subject.

### Cell viability

Neutrophil viability following 24 h rhERAP-treatment was determined through Trypan Blue exclusion assay. Briefly, 10 μL of cell suspension were mixed and incubated with 10 μL of 0.4% Trypan Blue (Merck-Sigma, Milan, Italy) in 96-well plates. Ten microliters of the mix were loaded on chamber slides and counted with the T20 Automated Cell Counter (Bio-Rad Laboratories, Hercules, CA, United States). Cells were routinely checked for *mycoplasma* contamination, and cell viability/count.

### Flow cytometry

At 24 h post rhERAP administration, cells were centrifuged (1,200 rpm for 10 min at RT) for 8 min, supernatants were stored at −80°C for further analysis while cells were washed 2 times with PBS and resuspended at the concentration of 5 × 10^5^ cells/100 μL of PBS incubated with the antibody for 15 min at room temperature (RT), protected by light. Neutrophils were characterized as CD15^+^ (Kromo Orange Bio-Legend) CD16^++^(Pacific Blue Beckman Coulter) CD45^dim^ (ECD Bio-Legend) expressing cells; neutrophil activation was assessed by the expression of MAC-1 (PC7 Bio-Legend), CD66b (APC Bio-Legend) and LAMP-1 (FITC Beckman Coulter) CCR7 (PC5.5 Bio-Legend). To verify ERAP1 and ERAP2 internalization neutrophils were incubated with anti-ERAP1 (R&D) 0.5 ug/100 uL + Goat anti-mouse Alexa Fluor and anti-ERAP2 (R&D) 0.5 ug/100 uL + Goat anti-mouse Alexa Fluor488 for 45 min. To assess ERAP internalization prior of antibody exposure neutrophils were treated for 45 min with BD’s Permeabilization buffer (cat 554715) and washed with BD’s Fix PERM buffer. After incubation with specific antibodies the cells were washed and immediately analysed by flow cytometry. The detection of intracellular granule cells during the last 6 h of stimulation, 1 μg/mL of Brefeldin A (Sigma-Aldrich) was added to block protein secretion. After 18 h neutrophils were permeabilized 30 min with the Fixation/Permeabilization Buffer (eBiosciences), stained with MPO (PE Beckman coulter), washed with PBS (Euroclone, Milan, Italy) and fixed in 1% paraformaldehyde (PFA, Sigma-Aldrich, MO, United States). Samples acquisition was performed on a CytoFLEX™ flow cytometer system equipped with CytExpert software (Beckman Coulter), and data were analyzed using Kaluza software, version 2.1.1. (Beckman Coulter).

### Cellular RNA extraction, reverse transcription, and gene expression in RT-PCR and ddPCR

Cellular RNA isolation, reverse transcription (RT) into cDNA, as well as amplification and quantification through real-time qPCR were performed according to a standardized protocol (Biasin et al., 2016). Briefly, 1 × 10^6^ cells were washed in PBS and collected in RNAzol^®^ (Duotech, Milano, Italy) and RNA extraction was performed through the phenol-chloroform method. RNA was dissolved in RNase-free water and quantified by the Nanodrop 2000 Instrument (1 μL, Thermo Scientific, Waltham, MA, United States). One μg of RNA was purified from genomic DNA with RNase-free DNase (RQ1 DNase; Promega) and reverse transcribed into first-strand cDNA with Moloney murine leukaemia virus reverse transcriptase along with random hexanucleotide primers, oligo dT, and dNTPs (Promega, Fitchburg, WI, United States). cDNA was amplified and quantified by real-time qPCR (CFX96 connect, Bio-Rad, Hercules, CA, United States) through SYBR Green PCR mix (Bio-Rad, Hercules, CA, United States), according to the following thermal profile: initial denaturation (95°C, 15 min), denaturation (15 s at 95°C × 40), annealing (1 min at 60°C) and extension (20 s at 72°C). The following genes were analysed: GAPDH, β-actin, NPRL3, GasderminD, IL-1 β, IL-8, CCL-2 and TNFα. Results for gene expression analyses were calculated by the 2^−ΔΔCT^ equation. Results are presented as the mean N fold (percent) ± SEM of the relative expression units to an internal reference sample and normalized to both the *GAPDH and β-actin* housekeeping genes.

Gene expression analyses of the main genes involved in the autophagy pathway (LAMP-1, LC3B, BCN1, TFEB, CHOP, XBP1, mTOR, SQSTM1/p62 and CASP3) were performed by droplet digital PCR (ddPCR QX200, Bio-Rad, Hercules, CA, United States). This analysis can increase the detection of rare transcripts and provide absolute quantification of RNA molecules. For all genes involved in the autophagy pathway, 3 µL of diluted cDNA (1:10.000) were mixed with specific primers and ddPCR EvaGreen SuperMix (Bio-Rad, Hercules, CA, United States). The mix was emulsified with droplet generator oil (Bio-Rad, Hercules, CA, United States) using a QX200 droplet generator, according to the manufacturer’s instructions. Droplets were transferred to a 96-well reaction plate and heat-sealed with a pierce able sealing foil sheet (PX1, PCR plate sealer, Bio-Rad, Hercules, CA, United States). PCR amplification was performed in a sealed 96-well plate using a T100 thermal cycler (Bio-Rad, Hercules, CA, United States). The thermal profile was: 3 min at 25°C, 60 min at 50°C, 10 min at 95°C, 45 cycles at 95°C for 15 s and at 60 s at 60°C, then 10 min at 98°C and finally hold at least for 30 min at 4°C. The 96-well plates were then transferred to a QX200 droplet reader (Bio-Rad, Hercules, CA, United States). Each well was queried for fluorescence to determine the number of positive events (droplets); the mRNA concentration was expressed as copies/uL. For ddPCR analysis, the QuantaSoft software version 1.7.4.0917 (Bio-Rad, Hercules, CA, United States) was used to quantify mRNA. Data are summarized as median and Interquartile (IQR) (25° and 75° percentile). All the primers were purchased as already optimized and used at 1x final dilution according to the manufacturer instructions (PrimePCR™ SYBR^®^ Green Assay, Bio-Rad).

### Cytokine and chemokine multiplex analysis

A 17-cytokine multiplex assay was performed on supernatants of neutrophil cell cultures stimulated with E1, E2 or E1+E2 using magnetic bead immunoassays according to the manufacturer’s protocol (BioRad, Hercules). Arbitrary concentrations of 4,000 pg/mL and 0.1 pg/mL were assigned to values respectively over or below the detection limit.

### Neutrophil migration assay

Cell migration was assessed using 3 μm pore Transwell system (Corning). Briefly, 0.5 × 10^6^ neutrophils were either untreated (CTR) or treated with 300 ng/mL of E1, E2 or E1+E2 for 24 h and then centrifuged. The supernatants were seeded in the lower chamber and used as chemoattractant, while cells were resuspended in RPMI 1640 + 10% FBS and seeded into the upper chamber of the Transwell insert. After 3h, migration was assessed by counting the migrated cells in the lower chamber with an automatic cell counter (Bio-Rad, Hercules, CA, United States) and stained for flow cytometry analyses. As the positive control (CTR+) of the migration assay, neutrophils were resuspended in RPMI+ 2% FBS, while in the bottom chamber control (CTR+) the medium was RPMI1640 + 90%FBS.

### Phagocytosis

Neutrophils (1 × 10^6^), stimulated as described above for 24 h, were cultured with latex beads coated with rabbit IgG–FITC complex (Cayman Chemical) to a final dilution of 1:100 for 1 h. Medium containing 0.05% Trypsin–EDTA (Code ECB3052D; EuroClone) was added for 10 min at 37°C in 5% CO_2_. Cells were then resuspended in RPMI 1640 supplemented with 10% FBS and centrifuged at 1,500 rpm for 10 min. Pellets were fixed with 0.1% PFA for 10 min, washed and resuspended in 50 μL PBS. Samples acquisition was performed on a CytoFLEX™ flow cytometer system equipped with CytExpert software (Beckman Coulter), and data were analysed using Kaluza software, version 2.1.1. (Beckman Coulter).

### ELISA

To quantify the release of granule proteins upon degranulation, supernatants from 24 h rhERAP-treated neutrophils were analysed via human MPO ELISA kit (eBioscience, Vienna, Austria), and human elastase ELISA kit (R&D system Minneapolis United States) according to the manufacturers’ instructions.

### Reactive oxygen species (ROS) production analysis

For the detection of ROS, 5 × 10^5^ neutrophils pre-treated with rhERAPs for 3 h were incubated with 10 µM of 2′–7′-dichlorofluorescein diacetate (DCFDA) (Thermo Fisher Scientific, Waltham, MA, United States) solution for 30 min at 37°C. DCFDA was deacetylated by cellular esterase to a non-fluorescent compound, which was then oxidized by ROS into the fluorescent molecule 2′,7′-dichlorofluorescein (DCF) (λexc = 495 nm, λemm = 529 nm). Dye fluorescent emission was measured at different time points (30, 60, 120 min) using a Varioskan LUX Multimode Microplate Reader (Thermo Fisher Scientific, Waltham, MA, United States). DCFDA fluorescence was normalized on nuclei after DAPI staining. Results are shown as the mean of three independent experiments performed in quintuplicate ± SD.

### Oxygen consumption measurements

Oxygen consumption rate triggered by ROS production by neutrophils was evaluated by high-resolution respirometry using the O_2_K oxygraph chambers (Oroboros, Instruments, Innsbruck, Austria). Granulocytes were trypsinized and 1 × 10^6^ cells were resuspended in the respiration medium MiR06 [0.5 mM EGTA, 3 mM MgCl_2_, 60 mM K-lactobionate, 20 mM taurine, 10 mM KH_2_PO_4_ 20 mM Hepes, 110 mM sucrose, and 1 g/L bovine serum albumin fatty acid-free, 280 U/mL catalase (pH 7.1)] and placed in the 2 mL closed oxygraphy and chamber at 37°C under continuous agitation (300 rpm). After 15 min of baseline O_2_ measurement, either rhERAPs (300 ng/mL) or PMA (100 ng/mL) were added. The concentration of O_2_ (µM) was monitored for 30 min and OCRs were expressed in pmol O_2_/(s*Million cells).

### Immunoblotting

Total proteins were extracted from 5 × 10^6^ cells using RIPA Buffer and protein concentration was measured with a BCA kit (MilliporeSigma) supplemented with 2% SDS, and a cocktail of protease inhibitors (Complete Roche Applied Science, Mannheim, Germany), sonicated, incubated on ice for 30 min on a platform rotator, and then centrifuged at maximum speed for 30 min at 4°C. Protein concentration from whole cell lysates was determined through BCA protein assay kit (Pierce, United States). Subsequently, 40ug of protein extracts were separated by 10% SDS-PAGE under reducing conditions (2.5% 2-mercaptoethanol) and were then transferred onto PVDF membranes. Membranes were blocked for 1 h with 5% bovine serum albumin (BSA) (Sigma-Aldrich, MO, United States) in Tris-buffered saline containing 0.01% Tween-20 (TBS-T), and probed overnight at 4 °C with the following primary antibodies: anti-SQSTM1 (p62) (Cell Signalling), 1:1000 in 5% BSA in TBS-T; anti-LC3B (Cell Signalling) 1:1000 in 5% BSA in TBS-T; anti-ERAP1 (R&D) 1:1000 in 5% BSA in TBS-T; anti-ERAP2 (R&D) 1:1000 in 5% BSA in TBS-T. After incubation with the appropriate HPR-conjugated secondary antibody (Cell Signalling) (1:10.000 in 5% BSA in TBS-T), immunoreactive bands were visualized by chemiluminescence (ECL, BioRad, Hercules, CA, United States). Quantification for all targets was performed through normalization to β-actin. For each experiment, a representative blot is shown, and the graphs show the mean ± SEM from n = 3 independent experiments.

### Immunocytochemistry

24 h post-rhERAP administration, 2.5 × 10^5^ neutrophils were washed in PBS and fixed with 4% PFA for 10 min at RT, followed by permeabilization with 0.1% Triton X-100 in PBS for 10 min at RT, blocking in 5% BSA in PBS for 1 h at RT, and incubation for overnight at 4°C in an humified chamber with primary antibodies Rabbit anti-LC3B, 1:1500 (Cell Signalling); Mouse anti-SQSTM1-P62, 1:300 (Cell Signalling), prepared in 1% BSA-PBS. The next day, samples were washed three times with PBS and incubated for 45 min at RT with secondary antibodies (Goat anti-mouse Alexa Fluor 488 (ab150113) or 647 (ab150115), or Goat anti-rabbit Alexa Fluor 488,647 (ab150079), 1:500, abcam, Cambridge, United Kingdom) prepared in 1% BSA-PBS. Negative controls were performed by omitting primary antibodies. After three washes in PBS, coverslips were carefully removed from the wells and mounted on Superfrost glass slides using a mounting medium with DAPI (Enzo Life Sciences, Milan, Italy). Confocal imaging was performed with a Leica TCS SP5 AOBS microscope system using a 20×/1.30 oil immersion objective (Leica Microsystems, Wetzlar, Germany).

### Statistical analysis

The Student’s t-test was done when appropriate for statistical analysis to compare continuous and categorical variables. One-way ANOVA or two-way ANOVA were applied for non-parametric comparisons. A *p*-value <0.05 was chosen as the cut-off for significance. Results are expressed as mean ± SEM from n = 5 independent experiments. All statistical analyses and graphs were performed with GraphPad Prism 9 (GraphPad Software, San Diego, CA, United States).

## Results

### Extracellular ERAPs are internalized in neutrophils without inducing cell toxicity

**FIGURE 2:**
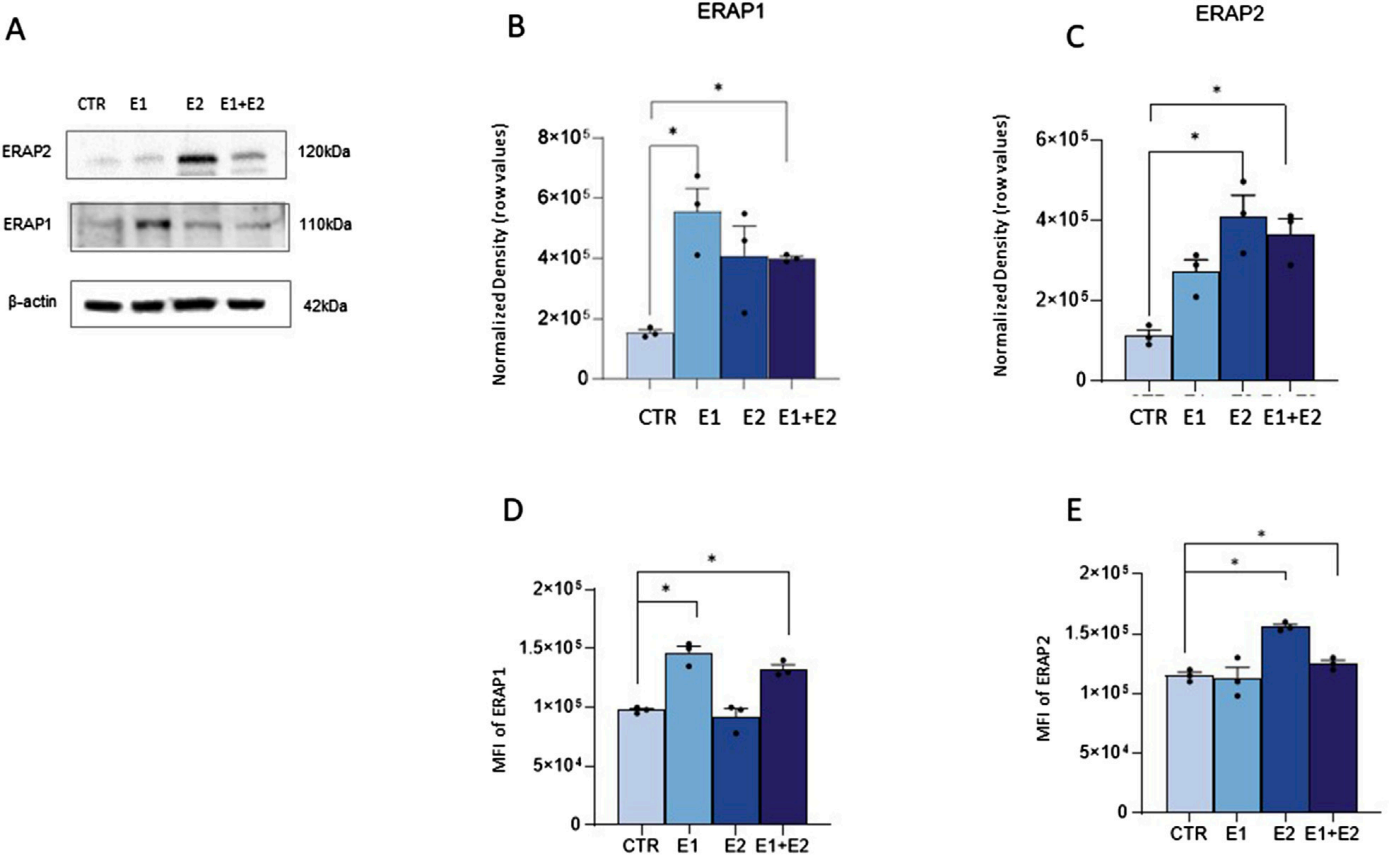
Neutrophils internalize exogenous ERAP1 and ERAP2 in the absence of cell toxicity. Representative Western blot for ERAP1,ERAP2 and β-actin in control (CTR) and rhERAP-exposed Neutrophils for 24 h **(A)**. Quantification of ERAP1 **(B)** and ERAP2 **(C)** normalized to β-actin. Neutrophils isolated from 3 healthy controls (HCs) were stimulated with 300 ng/mL of E1, E2, or E1+E2 for 24 h. Mean Intensity Fluorescence (MFI) neutrophil expressing the intracellular ERAP1 **(D)** and ERAP2 **(E)**. Values are shown as mean ± SEM. *p < 0.05. Results were analysed ANOVA *post hoc* Tukey test and *p* values were adjusted for multiple comparisons.

First, we confirmed the enzymatic activity of the rhERAP1 and rhERAP2 proteins (Supplementary Figure S1). To assess the internalization of these recombinant proteins, a Western blot analysis was performed on proteins extracted from neutrophils following 24 h treatment with 300 ng/mL rhERAPs. Western blot results (Figure 2A) show that neutrophils express a baseline concentration of both proteins (CTR) and effectively internalize E1 and E2 after 24-h of exposure. Indeed, increased levels of ERAP1 were detected in neutrophils treated with E1 and E1+E2 compared to CTR (*p* < 0.05 for both treatments) (Figure 2B). Likewise, ERAP2 levels increased in cells treated with E2 and E1+E2 compared to the control (*p* < 0.05 for both treatments) (Figure 2C). To further address this finding, we performed additional experiments using flow cytometry, and the results confirmed results from western blot analysis. Specifically, we marked extracellular and intracellular ERAP1 and ERAP2 separately. When cells were treated with recombinant E1 and E + E2, we observed an upregulation of ERAP1 intensity exclusively within neutrophils cells (CTR vs. E1 *p* < 0.05; CTRvsE1+E2 *p* < 0.05) (Figure 2D). Similarly, treatment with E2 and E1+E2 led to an increase of intracellular ERAP2 intensity (CTR vs. E2 *p* < 0.05; CTR vs. E1+E2 *p* < 0.05) (Figure 2E). Importantly, no fluorescence signals were detected in cells labelled with extracellular staining, indicating that the recombinant proteins were not merely bound to the cell surface but were indeed internalized (Supplementary Figure S2).

Notably, neutrophil viability assessed by an automatic cell counter, immediately and 24 h after rhERAP treatments was always over 90% in all conditions (data not shown).

### rhERAPs trigger neutrophil activation

**FIGURE 3:**
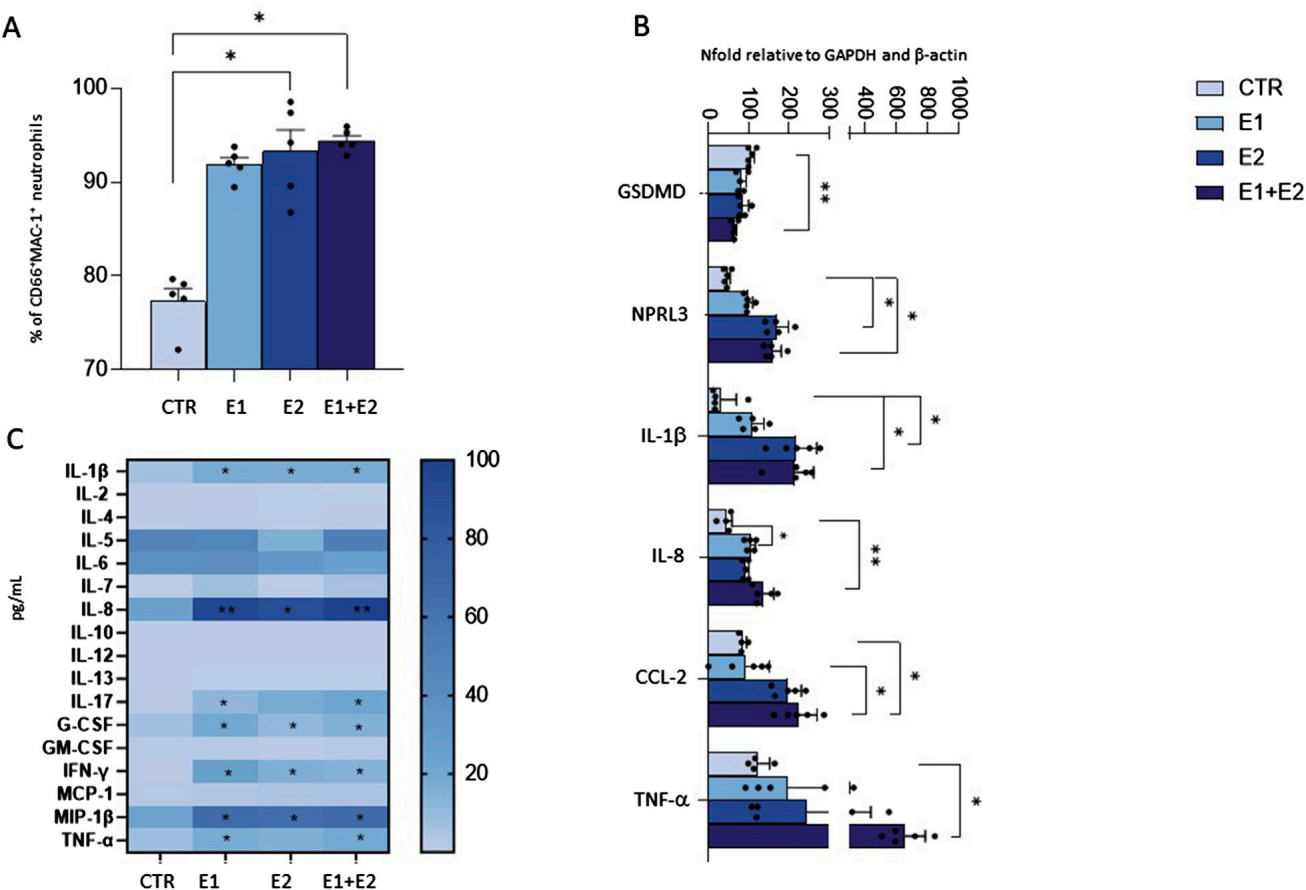
rhERAPs prompt neutrophil activation. Neutrophils isolated from 5 healthy controls (HCs) were stimulated with 300 ng/mL of E1, E2, or E1+E2 for 24 h. **(A)** The percentage of total CD45^dim^ CD15^+^ CD16^bright^ neutrophils expressing the activation markers MAC-1 and CD66b+ was assessed by flow cytometry. **(B)** mRNA expression of inflammatory determinants was evaluated 3 h post rhERAP administration by RT-QPCR. Values were normalized to GAPDH and are shown as mean ± SEM from 5 independent experiments. **(C)** The expression of 17-cytokines/chemokines was assessed by multiplex ELISA in supernatants from 24 h rhERAP-treated neutrophils of 5 independent experiments. Cytokine/chemokine productions (mean values) are shown as a color scale from white to blue (heatmap). Values are shown as mean ± SEM. *p < 0.05, **p < 0.01. Results were analysed ANOVA *post hoc* Tukey test and *p* values were adjusted for multiple comparisons.

To verify if rhERAPs impact neutrophil cellular homeostasis, we assessed neutrophil activation by flow cytometry and gene expression analyses. Results summarized in Figure 3A showed that the co-expression of the activation markers integrin Mac-1 (CD11b) and Carcinoembryonic antigen-related cell adhesion molecule 8 (CD66b) on neutrophils (CD15^+^ CD16^bright^ CD45^dim^) was significantly increased following E2 and E1+E2 stimulation compared to CTR (*p* < 0.05 for E2 and *p* < 0.01 for E1+E2). E1 treatment also boosted the expression of these activation markers, as well, although differences did not reach statistical significance.

Transcriptional analyses further confirmed that the addition of rhERAPs to the culture medium triggers neutrophil activation, as the expression of several genes orchestrating the inflammasome and cytokine signalling was enhanced. In detail, gene expression of targets involved in the canonical inflammatory milieu was significantly upregulated upon E1 (IL-8: *p* < 0.05), E2 (NPLR3: *p* < 0.05; IL-1β: *p* < 0.05) E1+E2 stimulation (NPRL3: *p* < 0.05; IL-1β: *p* < 0.05; IL-8: *p* < 0.01; TNFα: *p* < 0.05; CCL2: *p* < 0.05) compared to the CTR condition and E1 vs. E1+E2 (CCL2: *p* < 0.05) (Figure 3B). Conversely, GSDMD expression was significantly downregulated following E1+E2 stimulation compared to CTR (*p* < 0.01) (Figure 3B).

To further characterize the inflammatory profile, highlighted by gene expression analysis, the secretome of rhERAP-stimulated neutrophils was analyzed as well (Figure 3C). After 24 h of rhERAP stimulation, the concentration of IL-1β, G-CSF, IFN-γ, and MIP-1β was significantly increased in E1, E2, and E1+E2 compared to CTR (*p* < 0.05 for all comparisons). Notably, IL-8 production, the strongest chemoattractant for neutrophils, was augmented as well in rhERAP-stimulated cells (E1 and E1+E2: *p* < 0.001; E2: *p* < 0.05) (Figure 3C). As for IL-17 and TNF-α, statistically significant differences were observed only for E1 and E1+E2 compared to CTR (*p* < 0.05 for both comparisons) (Figure 3C). These results are in line with the pro-inflammatory profile observed in transcriptional analyses and indicate that following rhERAP stimulation, neutrophils release several cytokines, including IL-8, IL-17, TNFα and G-CSF, which are known to drive neutrophil’s migration, activation and recruitment. In addition, these initial results suggested that rhERAPs significantly affect neutrophil balance, pinpointing the need to further delve downstream into the molecular/cellular mechanisms.

### rhERAPs trigger neutrophil migration

We next investigated whether rhERAPs modulate neutrophil motility. To this end, we used a transwell migration model and counted migrated neutrophils while monitoring the expression of MAC-1, the most abundant and potent neutrophil adhesive/migration marker facilitating firm adhesion and transmigration of neutrophils through endothelium to site of infection and/or inflammation (Buffone et al., 2019).

**FIGURE 4:**
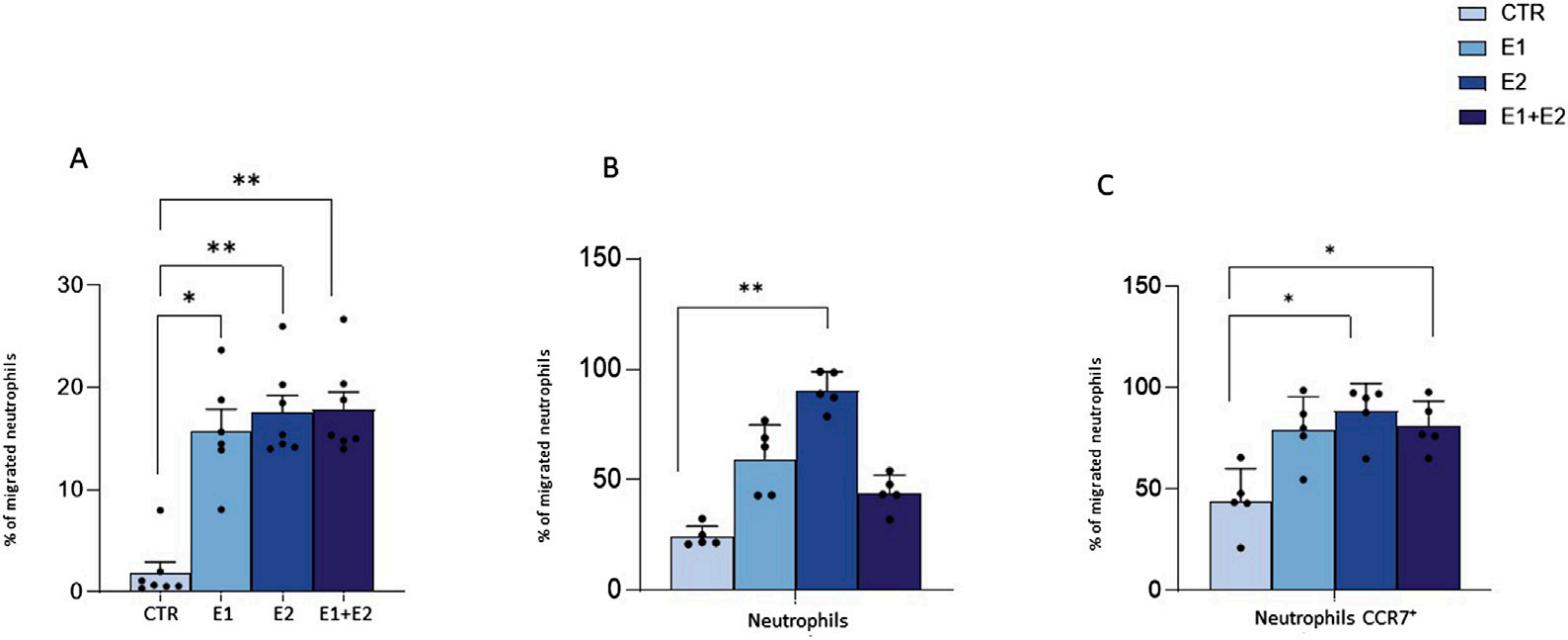
rhERAPs stimulates neutrophil migration. Transwell migration assays were performed to assess neutrophil migration 24 h after rhERAP administration. **(A)** Bars represent the mean percentage of cells recovered in the lower chamber compared to the number of cells added to the top chamber at the beginning of the assay. Each bar represents the mean of 5 independent experiments ±SEM. **(B)** The percentage of neutrophil migration was assessed by counting the CD15^+^ CD16^bright^ CD45^dim^ MAC-1^+^CD66b^+^ and CD15^+^ CD16^bright^ CD45^dim^ and **(C)** the CD15^+^ CD16^bright^ CD45^dim^ MAC-1^+^CD66b^+^ and CD15^+^ CD16^bright^ CD45^dim^ CCR7^+^percentage of neutrophils in the lower chamber. Values are shown as mean from 5 independent experiments± SEM. *p < 0.05, **p < 0.01. Results were analysed ANOVA *post hoc* Tukey test and *p* values were adjusted for multiple comparisons.

Results showed that neutrophil migration in the bottom chamber of the transwell containing conditioned medium from neutrophils that were 24 h pre-treated with E1, E2 and E1+E2, is significantly increased compared to the CTR condition (E2 and E1+E2 vs. CTR: *p <* 0.01; E1 vs. CTR: *p <* 0.05) (Figure 4A).

By using flow cytometry, we further characterized migrated neutrophils in the lower chamber of the transwell. Analysis performed on the basis of CD15^+^ CD16^bright^ CD45^dim^ revealed that most of the migrated cells are activated neutrophils, as assessed by MAC-1 and CD66b expression (E2 vs. CTR: *p <* 0.01) (Figure 4B). Furthermore, the expression of CCR7, a protein associated with migratory events, was significantly enhanced in the migrated neutrophils, compared to CTR (E2 and E1+E2 vs. CTR: *p <* 0.05) (Figure 4B). These data suggest that the extracellular milieu produced from rhERAP-exposed cells works as a chemoattractant for neutrophils.

### rhERAPs foster neutrophil phagocytosis

**FIGURE 5:**
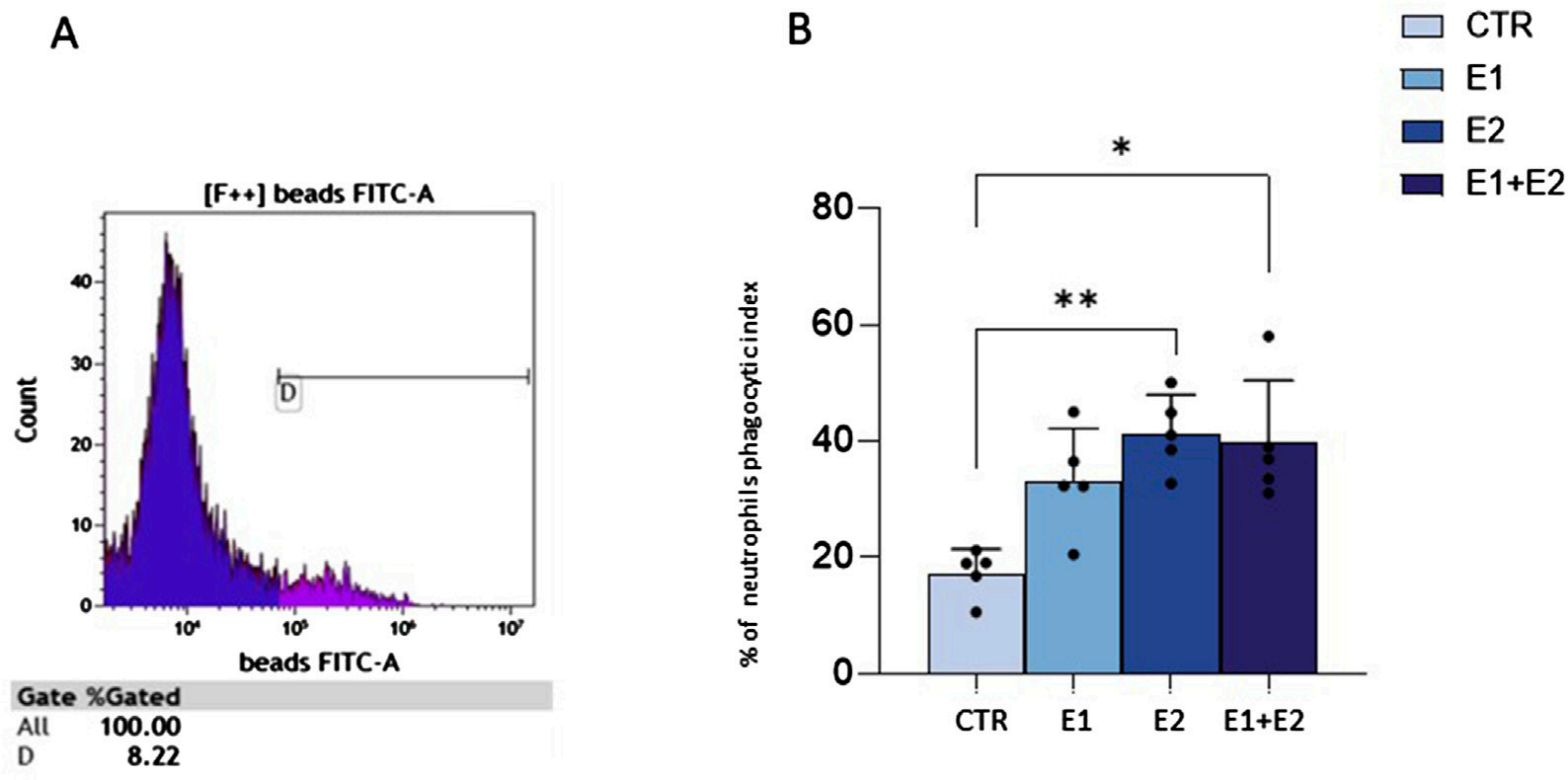
rhERAPs increase neutrophil phagocytosis. **(A)** Flow cytometry gating strategy in a CTR sample. Phagocytosis analysis relies on the fluorescence intensity of neutrophils. **(B)** The percentage of neutrophil phagocytic index was evaluated by flow cytometry 24 h post rhERAP-administration. Values are shown as mean of 5 independent experiments± SEM. *p < 0.05, **p < 0.01.

Since neutrophil migration is usually coupled with phagocytosis activation (Mayadas et al., 2014), we investigated if this cellular function could be induced by rhERAPs. According to the gating strategy on a CTR sample (Figure 5A), (representative flow cytometry plot (A) and gaiting strategy (B) reported in Supplementary Figure S3), data demonstrate that the ability of neutrophils to phagocytize latex beads coated with fluorescently labeled rabbit IgG is significantly increased following stimulation with rhERAPs (Figure 5B). In particular, stimulation with E2, and E1+E2 induced a significant increase (nearby 2-fold) in neutrophil phagocytic activity compared to CTR (E2 vs. CTR: *p* < 0.01; and E1+E2 vs. CTR: *p* < 0.05) (Figure 5B). E1-exposure also triggered neutrophil phagocytosis though differences did not reach statistical significance. Overall, these results indicate that extracellular ERAPs may contribute to enhancing neutrophil phagocytosis.

### rhERAPs trigger granule enzyme release by neutrophils

**FIGURE 6:**
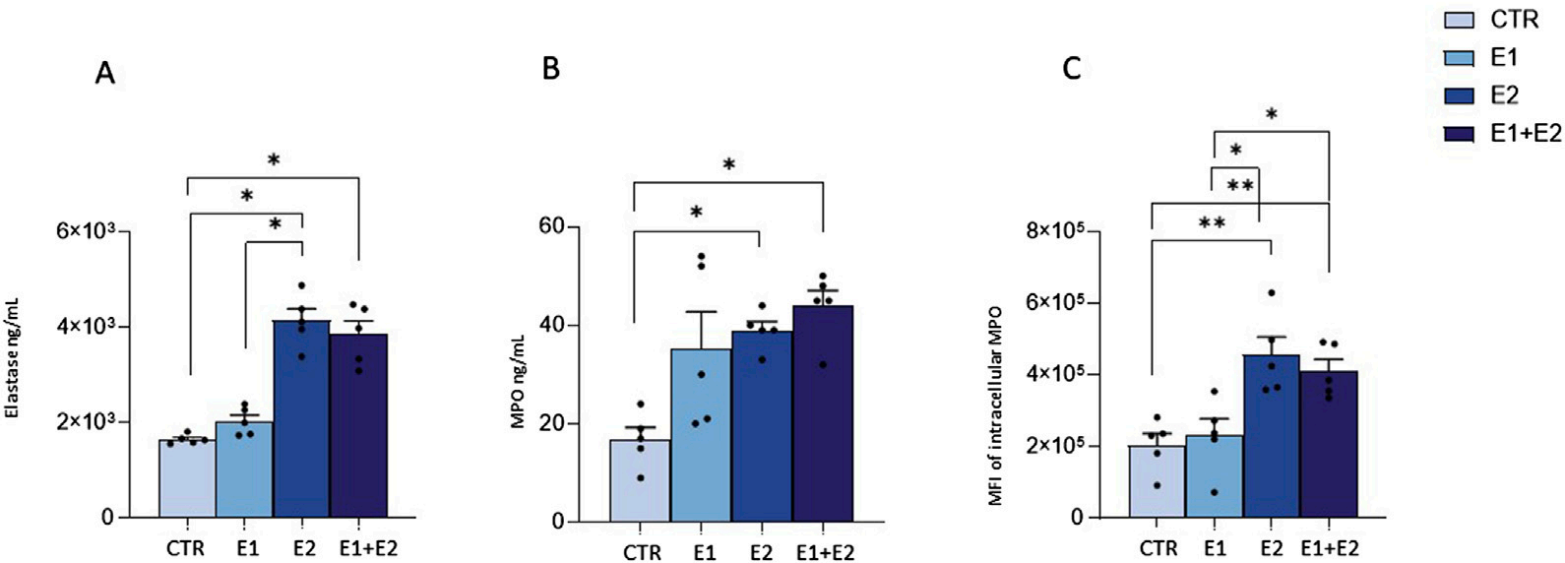
rhERAPs regulate neutrophil cytotoxic activity. **(A, B)** Neutrophils were cultured with 300 ng E1, E2 or both (E1+E2) for 24h, and elastase **(A)** and MPO **(B)** release was assessed by ELISA. **(C)** The percentage of neutrophil expressing intracellular MPO following rhERAP stimulation was assessed by flow cytometry. Values are shown as mean ± SEM. *p < 0.05, **p < 0.01. Results were analysed ANOVA *post hoc* Tukey test and *p* values were adjusted for multiple comparisons.

Besides phagocytosis, antimicrobial activity of neutrophils is based on the release of granules containing myeloperoxidase (MPO) and elastase into phagosomes (Rodríguez et al., 2003) or in the extracellular milieu (Voynow and Shinbashi, 2021; Forrest et al., 2022). Thus, we verified if rhERAPs could influence enzyme-containing granule exocytosis. Results showed this to be the case. Indeed, rhERAPs led to upregulation of elastase (E2 vs. CTR: *p* < 0.05; E1+E2 vs. CTR: *p* < 0.05; E2 vs. E1: *p* < 0.05) (Figure 6A) and MPO concentration in neutrophils’ supernatants (E2 vs. CTR*: p* < 0.05; E1+E2 vs. CTR: *p* < 0.05) (Figure 6B).

These results were further confirmed by flow cytometry analyses, as rhERAP-activated neutrophils (CD15^+^ CD16^bright^ CD45^dim^CD66b^+^MAC-1^+^) produced higher quantities of MPO compared to CTR (E2 vs. CTR: *p* < 0.01; E1+E2 vs. CTR: *p* < 0.01) (Figure 6C). In addition, E2 and E1+E2 treatments induced higher intracellular MPO production compared to E1 (E2 and E1+E2 vs. E1: *p* < 0.05). These findings suggest that rhERAPs optimize the antibacterial efficacy of neutrophils.

### rhERAPs reduce neutrophil ROS production without affecting oxygen consumption rate

**FIGURE 7:**
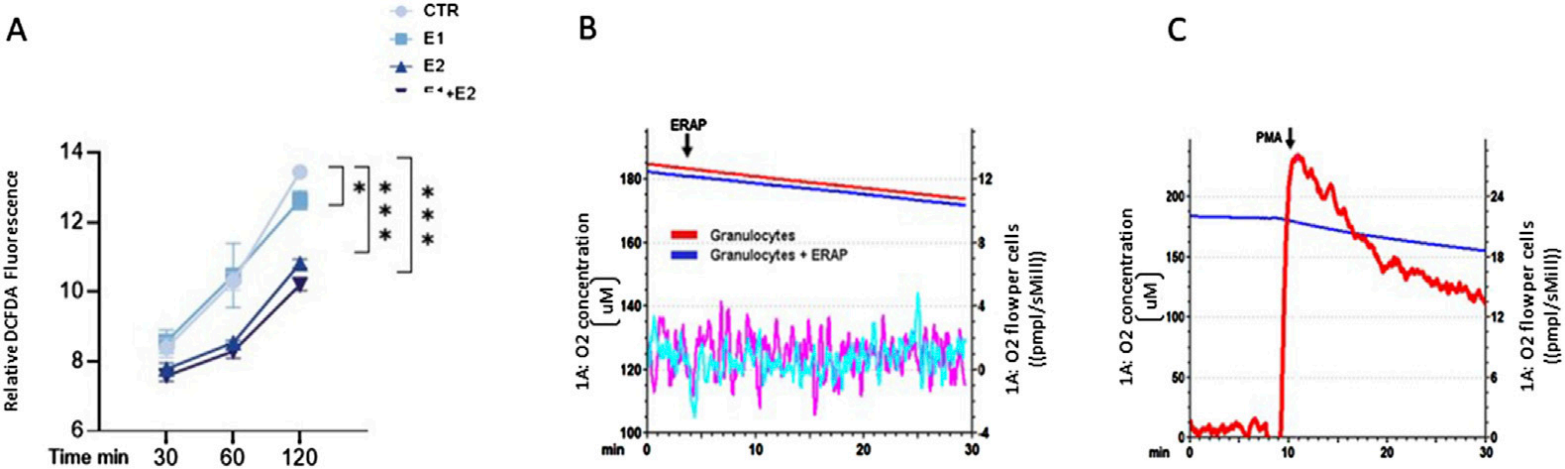
rhERAPs reduce ROS accumulation and do not induce oxidative burst. **(A)** Neutrophils were cultured with 300 ng E1, E2 or both (E1+E2) for 3h, and ROS production was measured by DCFDA assay. Fluorescence at 529 nm was measured. Results are reported as mean ± SEM from 5 independent experiments. *p < 0.05; ***p < 0.001. Results were analysed ANOVA *post hoc* Tukey test and *p* values were adjusted for multiple comparisons. **(B)** O_2_ concentration (μM) measurements of either untreated granulocytes or after rhERAP treatment (1 × 10^6^ cells) were monitored within an interval of 30 min. The arrow indicates the moment of rhERAP addition. Red and blue lines: oxygen concentration curves. Purple and cyano lines: derivative oxygen consumption fluxes normalized on cell number (1 × 10^6^ cells) (red-purple: untreated granulocytes; blue-cyano: rhERAP-treated granulocytes). **(C)** Baseline O_2_ consumption was evaluated for 10 min, then 100 ng/mL PMA was added to induce the oxidative burst. The arrow indicates the moment of PMA addition. Blue line: O_2_ concentration (μM); Red line: Derivative oxygen consumption fluxes normalized on cell number (1 × 10^6^ cells).

Given the link between neutrophil activation and reactive oxygen species (ROS) production, we investigated this pathway in rhERAP-stimulated neutrophils. Results obtained by DCFDA assay showed that E1, E2 and E1+E2 stimulations drastically reduce ROS intracellular accumulation, reaching statistical significance at 120 min post stimulation (E1 vs. CTR: *p* < 0.05; E2 vs. CTR: *p* < 0.001; E1+E2 vs. CTR: *p* < 0.001) (Figure 7A); these reductions are significant even when E2 and E1+E2 were compared to E1 (*p* < 0.05 for both treatments) (Figure 7A).

This is in line with the unchanged oxygen consumption rate (OCR) of neutrophils following rhERAP stimulation, measured by high-resolution respirometry (Figure 7B), in comparison to the ROS-releasing oxidative burst they undergo after phorbol myristate acetate (PMA) stimulation (Figure 7C).

### rhERAPs induce neutrophil autophagy

**FIGURE 8:**
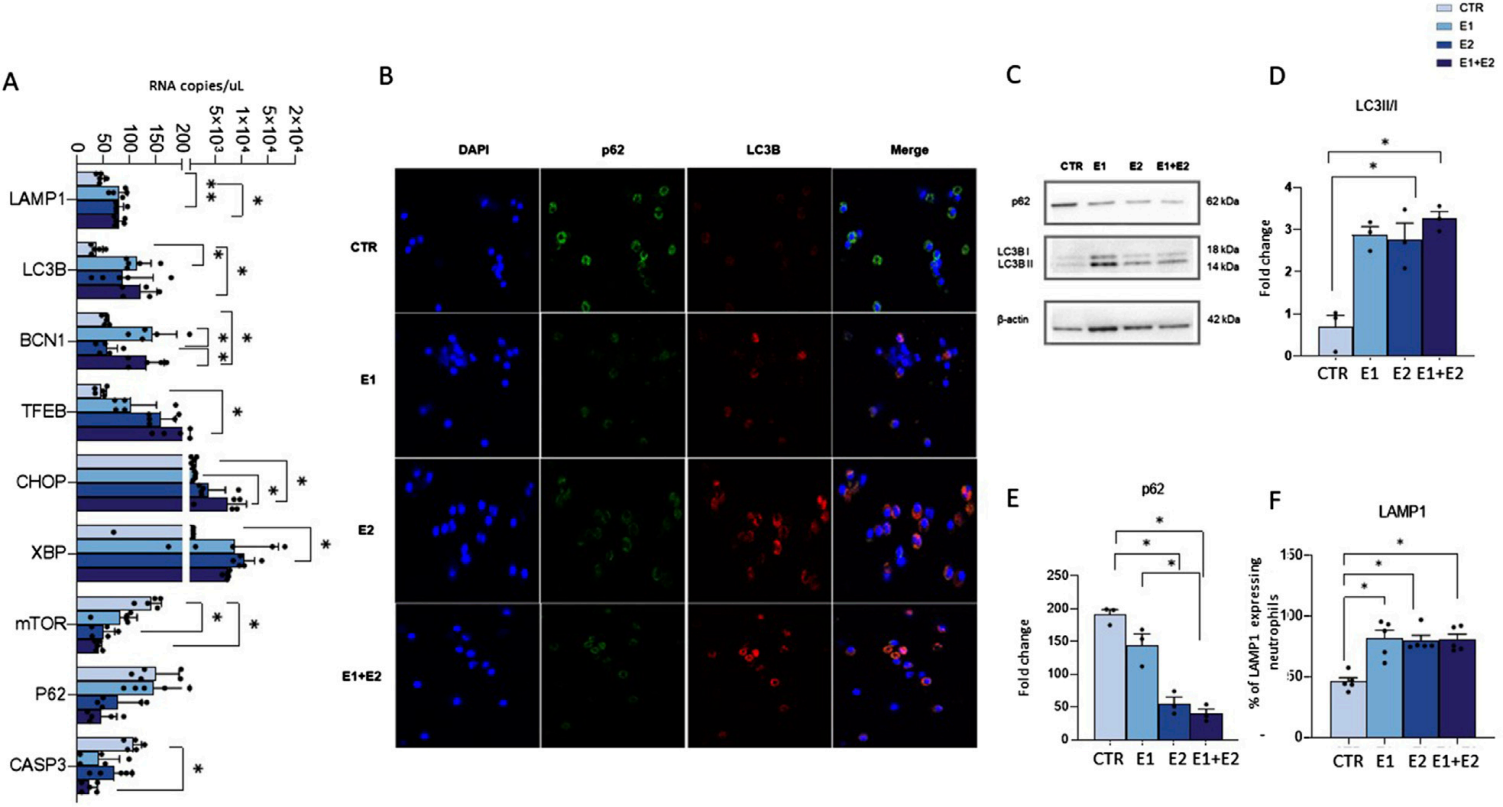
rhERAPs activate autophagy in neutrophils. **(A)** Autophagy-related genes were assessed by digital droplet PCR 3 h post rhERAP treatments and analysed. Results are expressed as mean ± SEM from 5 independent experiments (Unpaired *t*-test). **(B)** Representative image of immunofluorescence showing nuclei (DAPI blu) LC3B (red) and p62 (green) in CTR and rhERAP-treated neutrophils. **(C)** Representative Western blot against p62, and LC3B (LC3B-I and LC3B-II isoforms) in CTR and 24 h post rhERAP stimulation in neutrophil cell cultures. Histograms representing the densitometric quantification of LC3II/I ratio, **(D)** and p62 **(E)** in unstimulated (CTR), E1, E2 and E1+E2 stimulated neutrophils from 3 independent experiments. All targets were normalized on β-actin by Quantity One 4.6.6 and results are shown as normalized raw values corresponding to mean ± SEM. *p < 0.05. **(F)** Percentage of LAMP1-expressing neutrophils in unstimulated (CTR) E1, E2 and E1+E2 stimulated cells. Results are shown as mean ± SEM. *p < 0.05. Results were analysed ANOVA *post hoc* Tukey test and *p* values were adjusted for multiple comparisons.

Finally, as autophagy is implicated in the secretory pathway (Patel et al., 2013; Bhattacharya et al., 2014), we hypothesized that the upregulation of the lysosomal-mediated degranulation observed in rhERAP-stimulated cells could be linked to the modulation of autophagy by assessing mRNA expression (Figure 8A) and protein levels of autophagy-related markers (Figures 8C–F).

Results showed that rhERAPs early exposure (3 h) induce significant changes in the expression of genes that are related to the autophagy pathway either directly (BCN1, LC3B, SQSTM1/p62, TFEB, LAMP1, mTOR), or indirectly via the ER-stress-mediated Unfolded Protein Response (CHOP, XBP1, CASP3). Specifically, statistically significant differences were observed in E1 (BCN1: *p* < 0.05; LC3B: *p* < 0.05; LAMP1*: p* < 0.05; XBP1: *p <* 0.05), E2 (LAMP1: *p* < 0.01; TFEB: *p* < 0.05; XBP1: *p* < 0.05) and in E1+E2 stimulated cells (BCN1: *p* < 0.05; CHOP: *p* < 0.05; LC3B: *p* < 0.05; TFEB: *p* < 0.05; LAMP1: *p* < 0.05) compared to CTR. Finally, an increased expression of BCN1 was observed in E1 and E1+E2 vs. E2 (*p* < 0.05 for both treatments) and of CHOP in E1+E2 vs. E1 (*p* < 0.05). Notably, a drastic downregulation of CASP3, mTOR and p62 gene expression was observed in E2 compared to CTR for mTOR (*p* < 0.05) and in E1+E2 stimulated compared to CTR, reaching statistical significance for CASP3 (*p* < 0.05) and mTOR (*p* < 0.05) (Figure 8A). Immunofluorescence assay further confirmed autophagy activation in rhERAP-treated neutrophils. Indeed, as shown in the representative Figure 8B, E1E2E1+E2 stimulations resulted in a qualitative accumulation of LC3B along with p62 reduction (Figure 8B).

Finally, western blot analyses corroborated these results (Figure 8C). Indeed, compared to CTR, an increase in LC3B-II/I (E2: *p* < 0.05; E1+E2: *p* < 0.05) (Figure 8D), and a decrease of p62 (E1, E2 and E1+E2: *p* < 0.05) (Figure 8E) were observed in rhERAP-stimulated cells. These data suggest that rhERAP exposure increases the availability of the lipidated LC3 isoform for autophagosome formation and effective degradation of p62 protein, supporting an effective progression of the autophagy pathway. Notably, this was coupled with a reduction of mTOR mRNA and an increase in LAMP1 protein as assessed by flow cytometry (E1; E2; E1+E2 vs. CTR: *p* < 0.05 for all comparisons) (Figure 8F), consistent with the transcriptional upregulation of LAMP1 mRNA. These data further support the implication of rhERAP-induced autophagy in lysosomal-mediated neutrophil degranulation possibly via ER stress and mTOR inactivation.

## Discussion

Several reports have shown that, besides antigen processing, ERAP1 and ERAP2 may be released into the extracellular milieu following inflammatory stimuli, activating macrophages as well as natural killer cells (Goto et al., 2014; Goto et al., 2015; Goto et al., 2018; Saulle et al., 2019). Such findings indicate that ERAPs play a significant role in both innate and acquired immunity and justify the observation that ERAPs dysregulation can result in the onset and/or progression of several diseases including autoimmune illnesses (Babaie et al., 2020; Reeves et al., 2020; Limanaqi et al., 2023), tumors (Dhatchinamoorthy et al., 2021; Temponeras et al., 2022; Schmidt et al., 2023) and infections (Biasin et al., 2013; Liu et al., 2017; Steinbach et al., 2017; Reeves et al., 2019; Saulle et al., 2019; 2020a; 2020b; Klunk et al., 2022). Considering the prominent role of neutrophils in all of these diseases, investigating the connection between ERAP1/ERAP2 and neutrophil regulation is particularly pertinent.

In this study, by adding rhERAP1 and rhERAP2 to neutrophil cell culture supernatant to mimic their stressor-induced secretion, we found that these proteins modulate: 1) neutrophil activation by increasing the expression of specific markers as well as the transcription/release of inflammatory factors; 2) migration; and 3) microbial killing by boosting phagocytosis, autophagy, MPO and elastase release. Moreover, we found that rhERAPs reduce ROS accumulation in neutrophils, possibly to prevent their death and/or damage.

To our knowledge, this is the first study investigating the intricate relationship between ERAP1, ERAP2 and neutrophils, shedding light on the molecular interplay that modulates neutrophil functions. The only previous studies linking ERAPs and neutrophils were performed in the setting of Behçet’s disease (BD). In particular, it was demonstrated that in BD, some ERAP1 SNPs favour the generation of autoantigens that, in turn, trigger CD8^+^ T cells to release cytokines (IL-17, IL-8, and GM-CSF) which activate neutrophils (Khoshbakht et al., 2023). Contrary to our findings, Hye-Myung and colleagues reported a higher frequency of CD11b^+^ Ly6G^+^ neutrophils in an ERAP1^+/−^mouse model of HSV-induced BD, suggesting that a reduced expression of ERAP1 enhances neutrophil infiltration (Ryu et al., 2023). The absence of ERAP2 in the mouse genome (Andrés et al., 2010) as well as the pathological context of BD could at least partially justify the discrepancies reported in this and our study, but further analyses are needed to dissect the role of rhERAPs on neutrophil recruitment/activation.

The involvement of ERAPs in chemotaxis has already been documented. In particular, Sato Y. demonstrated that ERAP1 plays a crucial role in VEGF-stimulated proliferation and migration of endothelial cells, as well as angiogenesis, via the binding and modification of PDK1 (Akada et al., 2002; Miyashita et al., 2002; Yamazaki et al., 2004; Suzuki et al., 2007). Moreover, ERAP2 knockdown was shown to weaken the capacity of PSCs to promote migration and invasion of pancreatic cancer cells (Guan et al., 2022). The induction of neutrophil migration by rhERAP is, however, a novel observation. The driving factor in this process is probably represented by IL-8, which is known to be a chemotactic determinant for neutrophils (Metzemaekers et al., 2020). Indeed, IL-8 production by rhERAP-treated monocyte derived macrophages (MDM) was reported in a previous work (Saulle et al., 2019) and further confirmed in this study following rhERAP neutrophil stimulation. However, additional studies are warranted to confirm these results and to better understand the molecular mechanisms by which secreted ERAP1 and ERAP2 could modulate neutrophil migration, as it would open new avenues for exploring their therapeutic potential in settings characterized by altered cell motility, such as autoimmune diseases and cancer metastasis.

Autophagy, a cellular process with a pivotal role in maintaining cellular homeostasis (Gómez-Virgilio et al., 2022), is increasingly recognized as a central regulator in various aspects of neutrophil biology. Indeed, autophagy facilitates the selective targeting and engulfment of intracellular pathogens (Riebisch et al., 2021), intervenes in supporting antitumor immunity (Jiang et al., 2021), and, by promoting cellular balance, it contributes to immune tolerance and helps to prevent the activation of autoreactive immune responses (Xia et al., 2021). Yet, several studies also suggest that autophagy may cause disease, thus acting as double edge sword (White and DiPaola, 2009). The nexus between ERAPs and autophagy has been analysed in more than one study (Kenna et al., 2015; Thomaidou et al., 2020; Nakamura et al., 2021; Blake et al., 2022b; Wang et al., 2022). Definitely, an aberrant peptide processing may trigger the accumulation of unstable protein complexes in the ER, a phenomenon that drives endoplasmic reticulum–associated protein degradation (ERAD) and UPR, which in turn, activates autophagy (Nakamura et al., 2021; Blake et al., 2022b; Wang et al., 2022).

More recently, ERAP2 was demonstrated to play an important role in autophagy of PSCs via a UPR-signaling pathway (Guan et al., 2022). Indeed, silencing of ERAP2 in PSC promotes a quiescent state and a drastic reduction of UPR mediated autophagy as assessed through calnexin, IRE1a, PERK, LC3II lipidated form, and p62 degradation analyses (Guan et al., 2022). In addition, both ERAP1 and ERAP2 were found to be charged within autophagic vesicles (Schmitt et al., 2022). Our results suggest that rhERAP treatments induce ER stress response as reflected by the upregulation of ER stressor genes CHOP and XBP1. This in turn, promotes the autophagy/lysosomal pathway activation as shown by increased LC3II/I ratio, parallelled by a decrease in p62 protein along with mTOR mRNA downregulation. Our results are in line with a previous study published by Wang and colleague’s. They demonstrated that PBMCs isolated from Taiwanese with Ankylosing Spondylitis carrying the ERAP1-001 haplotype, leading to ERAP1 overexpression, display higher production of β2m-free heavy chain (FHCs) and FHC dimers, UPR (BiP, CHOP and XBP1), autophagy (Beclin-1, LC3 I and LC3 II) and inflammatory (caspase 1 and IL-1β) markers, compared to ERAP1-002 homozygous donors, who produce lower ERAP1 quantity (Wang et al., 2022). In another study on U937 the overexpression ERAP1 activates in cascade misfolding of HLA-B27 complexes, UPR, and autophagy, that by reducing ER stress may offer cytoprotective benefits (Wang et al., 2022). Further supporting this theory, in HLA-B27 transgenic rats Tran et al. observed an increased folding of this MHC class I molecule following ERAP1 knockdown, which in turn was associated with lower intracellular accumulation of FHC dimers as well as BiP, CHOP and XBP1 expression suggestive of reduced UPR (Tran et al., 2023). Overall, the control of autophagy by reducing ER stress could therefore provide cytoprotection (Chipurupalli et al., 2021) even in neutrophils. This finding holds potential for setting up targeted therapeutic interventions in conditions where autophagy dysregulation and ERAPs are implicated.

Unexpectedly, findings from this study also demonstrated that rhERAPs significantly reduce ROS production by neutrophils. Although a modest increase of ROS leads to enhanced cell proliferation, survival and protective immune responses (He et al., 2021), ROS stress overwhelming cellular antioxidant capacity can damage nucleic acids, proteins and lipids, favouring the progression of inflammatory disorders (Vona et al., 2021). In the context of rhERAP-induced neutrophil activation and cytotoxic activity, we would have expected a partial increase in ROS production. Accordingly, Nikopaschou et al. (2024) recently provided compelling evidence that in e A375 cancer cell lines, blocking ERAP1 function -either through the use of specific inhibitors or via genetic silencing - resulted in a substantial decrease in ROS production. The significant drop observed in our study is, therefore, intriguing and might represents a compensatory protective mechanism of neutrophils to prevent cellular dysfunction and tissue injury. Moreover, the potential molecular determinants partaking in the regulation of this complex pathway should be identified and investigated to clarify this unexpected result.

T lymphocytes were not present in our *in vitro* experimental model; we therefore assume that the activation of cellular pathways in neutrophils is not a secondary response driven by acquired immunity. Moreover, although Western Blot and flow cytometry analyses suggest an internalization of rhERAP proteins into neutrophils, the adopted techniques do not allow to distinguish between exogenous and endogenous proteins. Further analyses, using tagged proteins should be used to clarify this issue and provide information regarding the subcellular localization of the endocytosed protein. At this stage, we cannot rule out that the above-described remodelling of neutrophil homeostasis partially relies on the trimming of molecules in the extracellular milieu, contributing to the activation of still unidentified signalling cascades in neutrophils.

Overall, our study demonstrated that, although E1 and E2 were expected to exhibit complementary or additive effects in combination treatments, results indicate that their effects on neutrophil activation, migration, and autophagy are not additive. Though heterodimerization of ERAP1 and ERAP2 has been reported to enhance peptide cleavage efficiency and antigen presentation (Evnouchidou et al., 2014), it is unclear whether this additive effect could also extend to other cellular processes, such as autophagy. Moreover, in our experimental design, the combination treatment involved half the concentration of E1 and E2 compared to the single aminopeptidase treatments. This adjustment may have contributed to the lack of observable additive effects, as the combined concentration might not reach the threshold necessary for potentiating the analysed pathways. Finally, although, E2 consistently demonstrates more pronounced effects on the examined cellular processes, we cannot exclude the possibility that these differences are influenced by the different substrate specificity of the two enzymes (Kloetzel and Ossendorp, 2004) or the recombinant nature of the employed proteins. Artificial recombinant proteins may exhibit variations in stability, folding, or receptor interactions, which could impact their biological effects. Further studies using native proteins or alternative production methods may be necessary to disentangle these possibilities and confirm whether the observed effects reflect true biological features.

We acknowledge that our findings are primarily descriptive and observational. In this exploratory study, we did not use specific genetic or pharmacological modulators to target the various pathways analysed or to regulate ERAP activity, which would have allowed us to confirm that the effects induced by ERAP are directly linked to their stimulation/suppression and/or their enzymatic activity. This limitation is largely due to the complexities of working with a limited number of primary cells. However, in future research, we plan to incorporate these strategies, so as to gain a clearer understanding of the underlying mechanisms behind the phenomena observed in this study.

**FIGURE 9:**
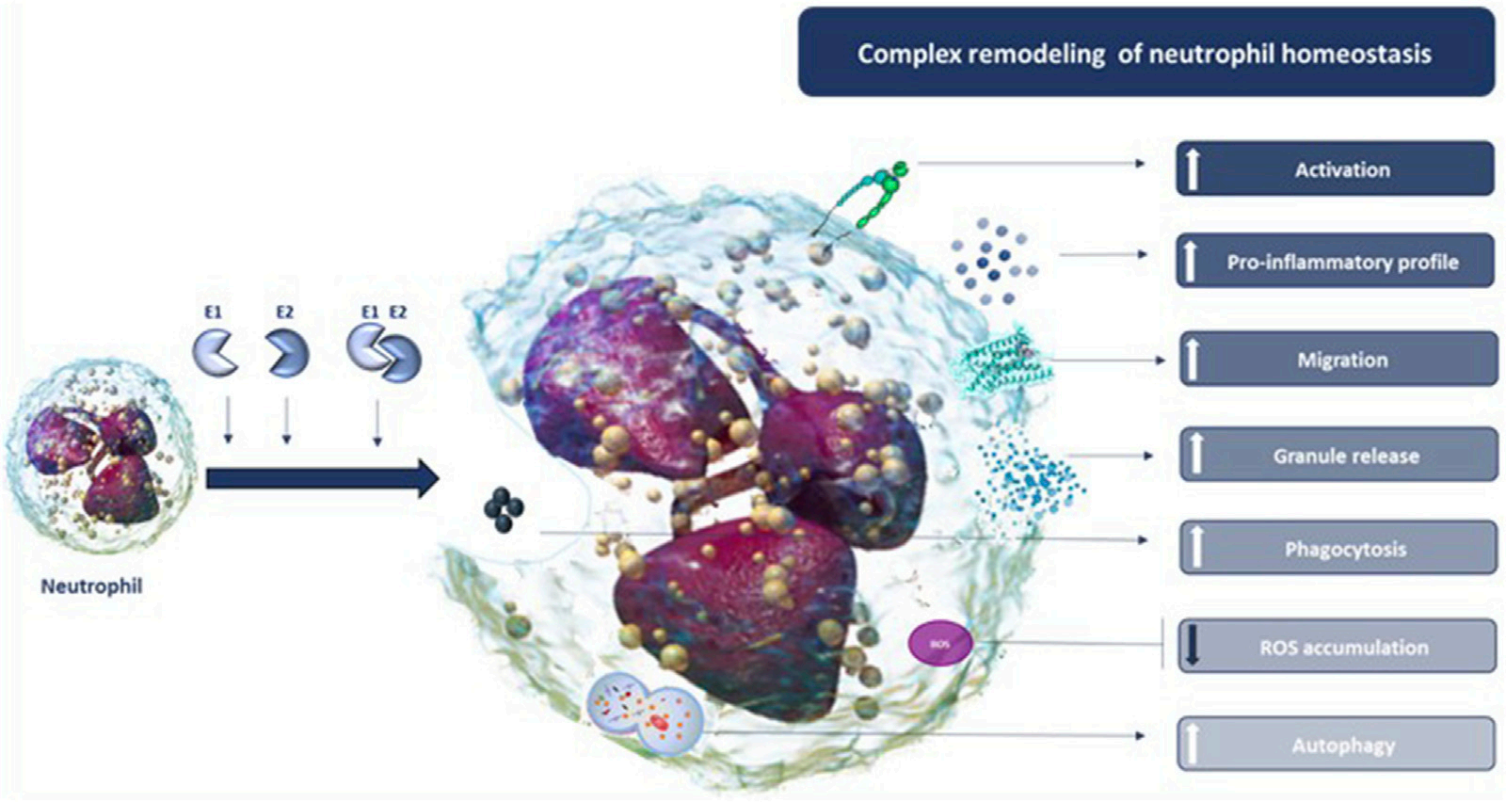
Outline of cellular remodeling mechanisms induced by rhERAPs on neutrophil homeostasis. Summarizing cartoon of the main cellular mechanisms modulated by rhERAP-exposure in neutrophils.

However, data gathered in this study and summarized in Figure 9 show an exhaustive overview and suggest that rhERAPs work as robust modulators of biological neutrophil functions, implying that stressor-induced ERAP-secretion could have direct and/or indirect effects on neutrophil activation.

As neutrophils are the most abundant white blood cell population in the circulation (Granot, 2019) and they orchestrate complex functions in many clinical scenarios, these cells could serve as a target for therapeutic approaches. Identification of molecules such as rhERAPs, able to modulate neutrophils and activate or block specific neutrophil functions, including ROS accumulation, could thus be useful to circumvent hurdles associated with utilizing neutrophils for therapeutic purposes (Kirsten et al., 2015; Lazaar et al., 2020).

The intricate relationship between ERAP1, ERAP2, and neutrophils highlights the expanding role of these aminopeptidases beyond antigen processing and presentation. Understanding the molecular mechanisms governing their interaction with neutrophils provides valuable insights into the regulation of innate immune responses. Further validations in preclinical animal models are, nevertheless, warranted to determine the translational relevance of our findings in more physiologically complex and relevant contexts, ensuring a scientifically rigorous approach before proposing any therapeutic intervention for ERAP-dependent diseases.

## Data Availability

The raw data supporting the conclusions of this article will be made available by the authors, without undue reservation.

## Supplementary material

SUPPLEMENTARY FIGURE S1 rhERAPs retain enzymatic activity. **(A)** Enzymatic activity of ERAP1 and **(B)** ERAP2 was measured by their ability to cleave the fluorogenic peptide substrate, Arg¬7¬amido-4¬methylcoumarin (Arg¬AMC). Fluorescence was read to 460 nm in kinetic mode for 5 min.

SUPPLEMENTARY FIGURE S2 Gating strategy for ERAP2 expression in neutrophils after rhERAP2 administration by flow cytometry. The gating strategy began with the exclusion of doublets and dead cells in the first gate **(A)**. Subsequently, neutrophils were identified in **(B)** by selecting CD15-positive cells, and ERAP2 expression was analyzed within this population. **(C)** displays a histogram representing the CD15-positive population and their ERAP2 expression. To control for false positives, a secondary antibody-only control was used **(D)**, and the same gating (Gate B) was applied across all samples, from the negative control to treated cells, to accurately quantify the percentage of positivity. Finally, **(E)** illustrates the extracellular staining of neutrophils for ERAP2.

SUPPLEMENTARY FIGURE S3 Representative flow cytometry plots for data reported in Figure 5B. Neutrophils were selected as CD66b^+^ CD15^+^ CD45^dim^, and the phagocytosis of IgG-latex beads-FITC was analyzed. Representative flow cytometry plots for control **(A)** and following E1 **(B)**, E2 **(C)**, and E1+E2 **(D)** administration are reported.
