# Supplementary figures and images for: Impact of endoplasmic reticulum aminopeptidases 1 (ERAP1) and 2 (ERAP2) on neutrophil cellular functions
Source: Front Cell Dev Biol. 2025 Jan 7;12:1506216. doi: 10.3389/fcell.2024.1506216 (PMC11747162; doi:10.3389/fcell.2024.1506216)

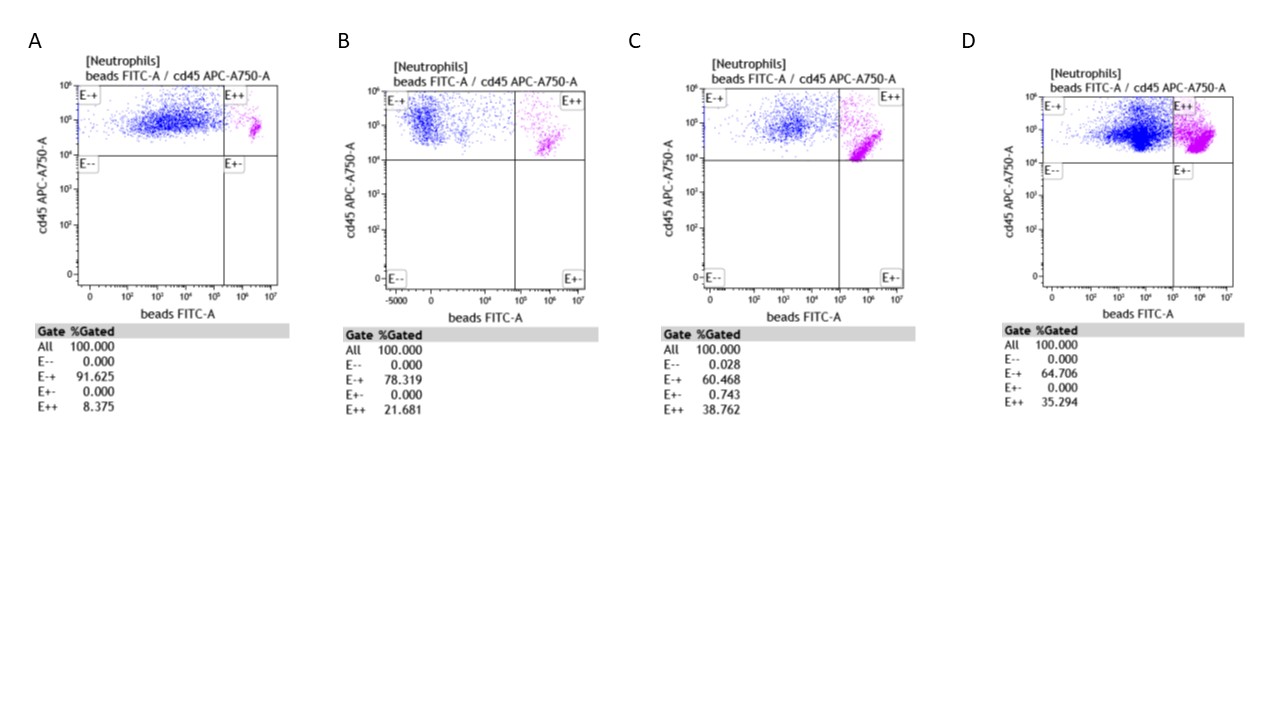

Supplement: Supplementary file 1 [file Image3.jpeg]

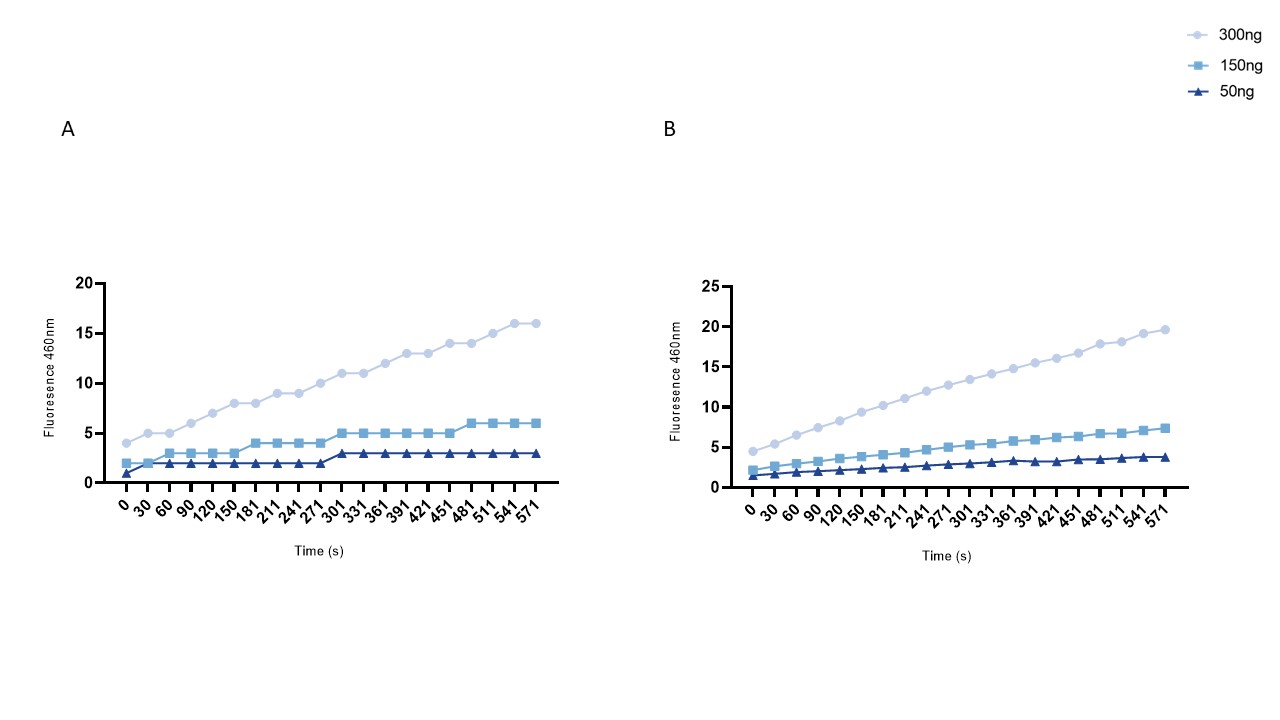

Supplement: Supplementary file 2 [file Image1.jpeg]

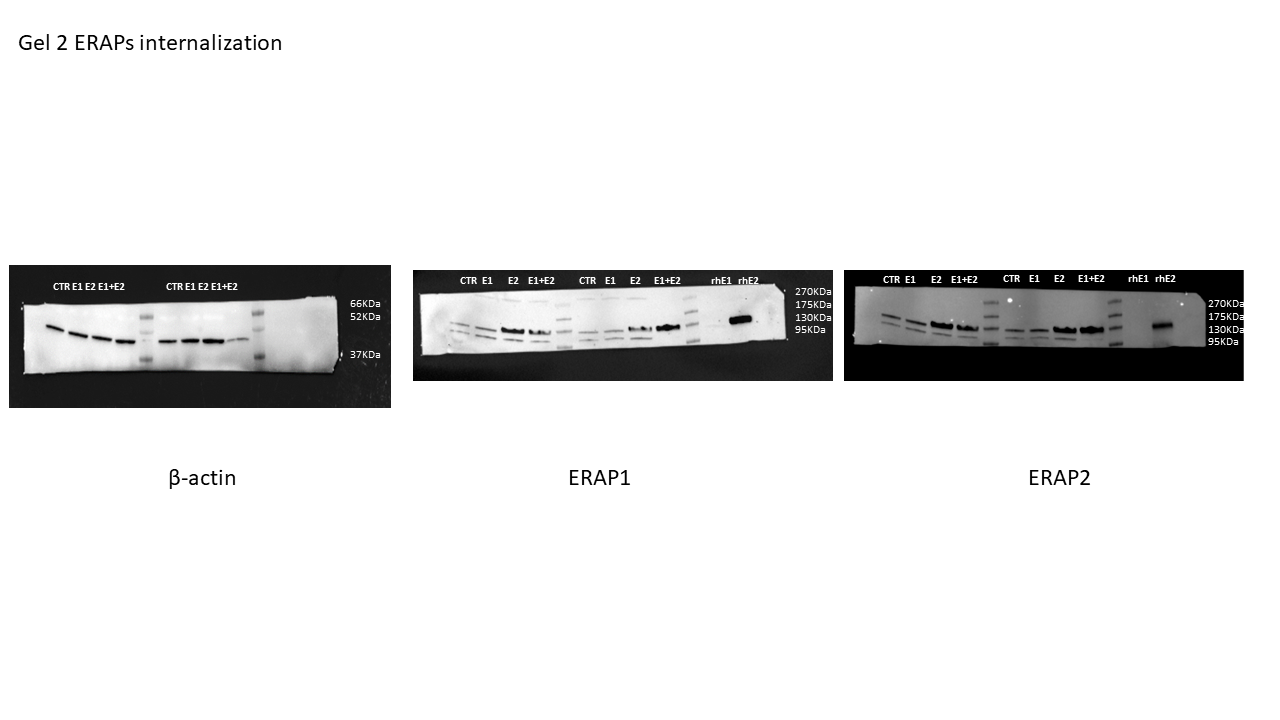

Supplement: Supplementary file 3 [file DataSheet1.zip › immagini/gel 2 ERAP internalization.tif]

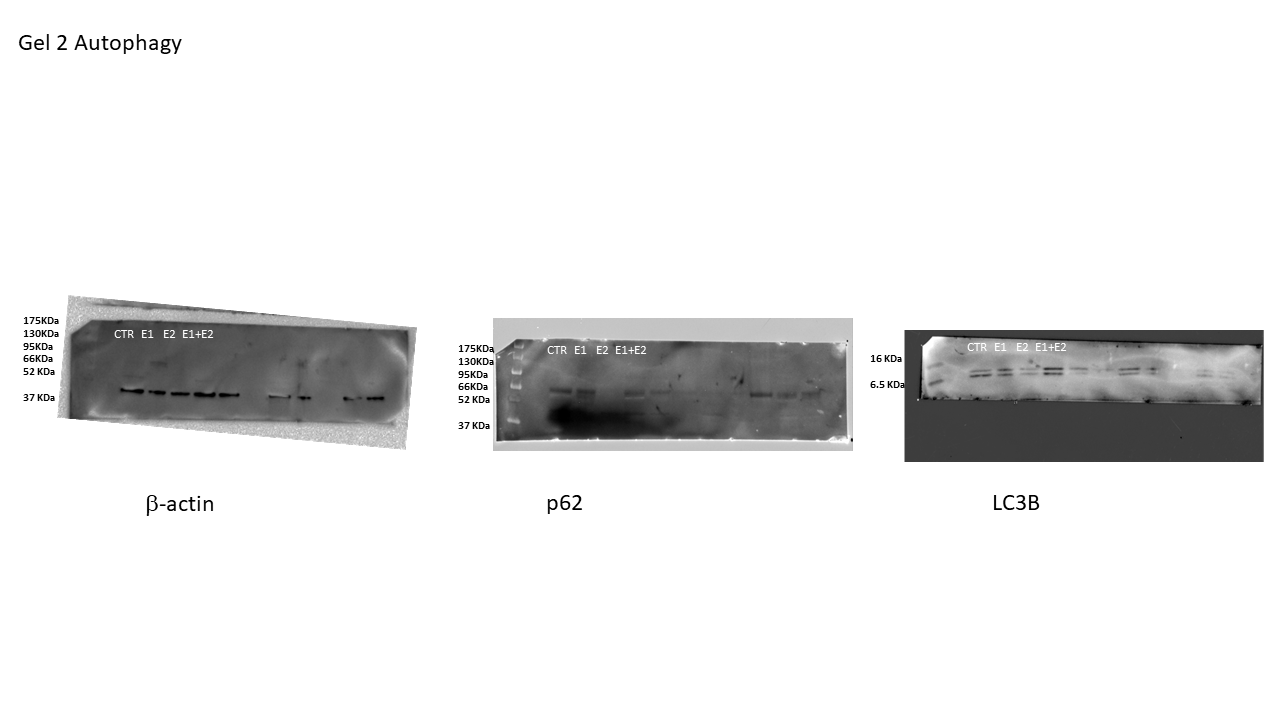

Supplement: Supplementary file 3 [file DataSheet1.zip › immagini/gel 1 autophagy.tif]

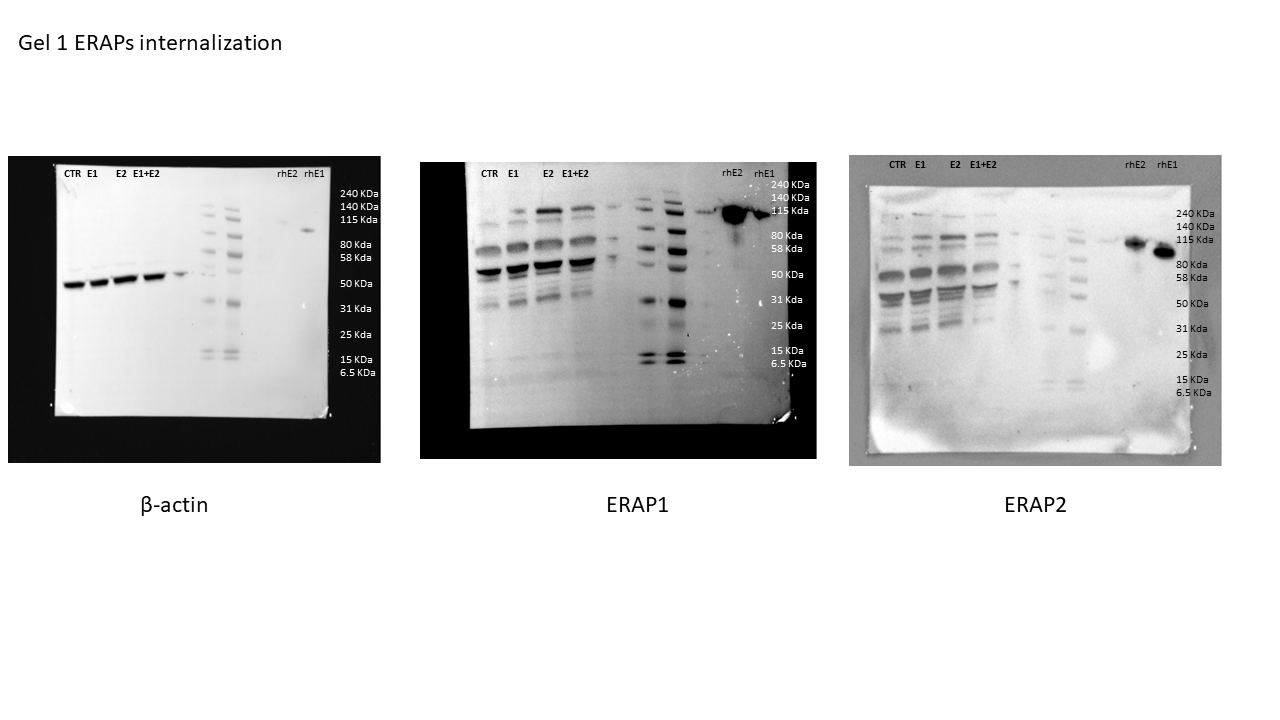

Supplement: Supplementary file 3 [file DataSheet1.zip › immagini/gel 1 ERAP internalization.tif]

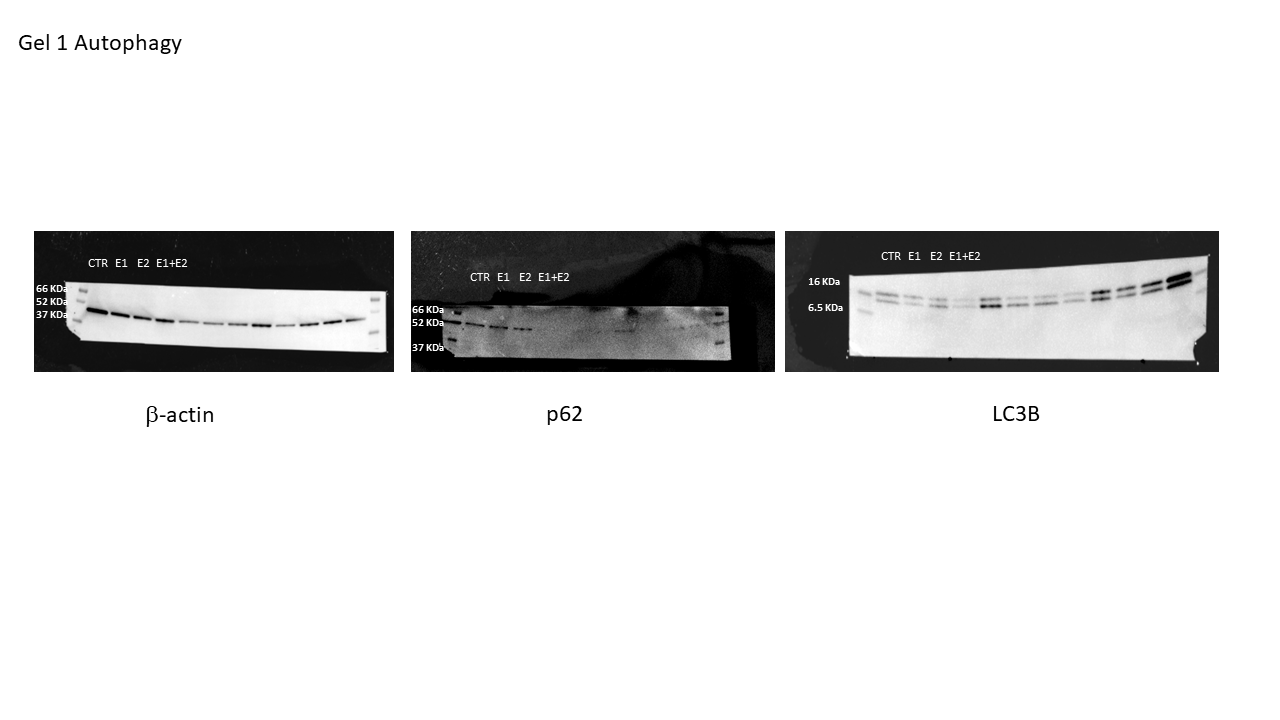

Supplement: Supplementary file 3 [file DataSheet1.zip › immagini/gel 2 autophagy.tif]

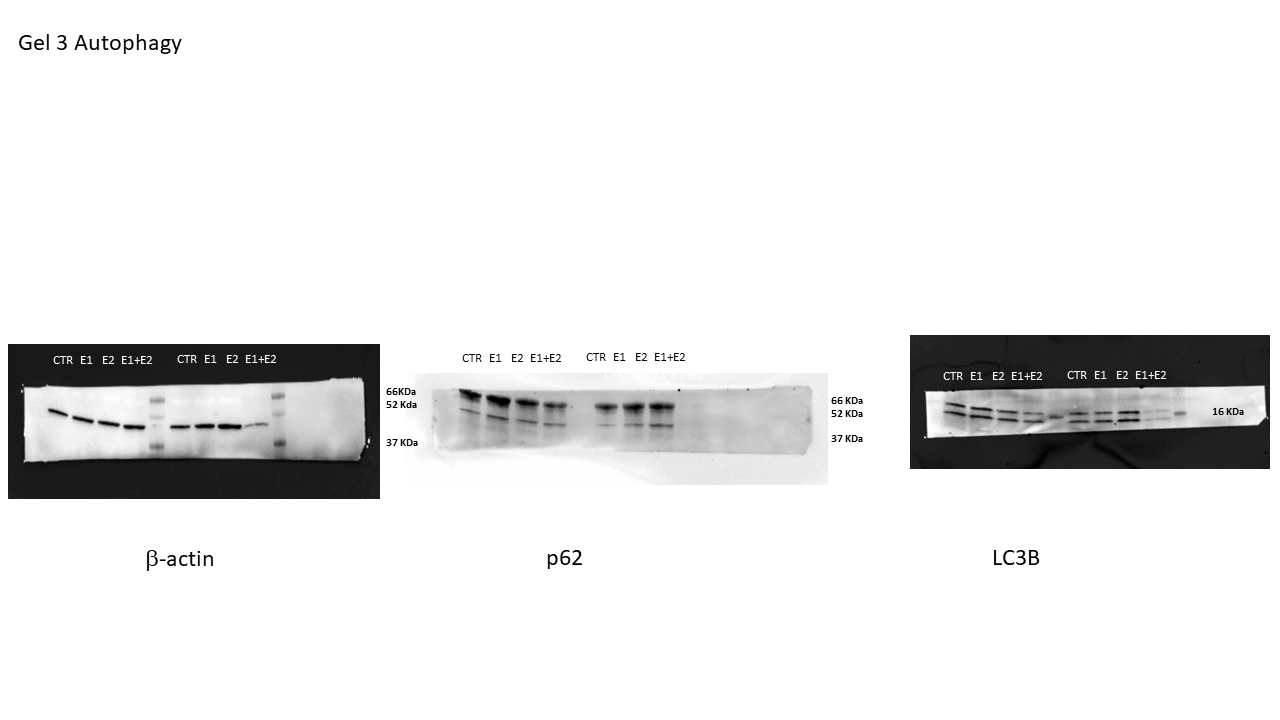

Supplement: Supplementary file 3 [file DataSheet1.zip › immagini/gel 3 autophagy.tif]

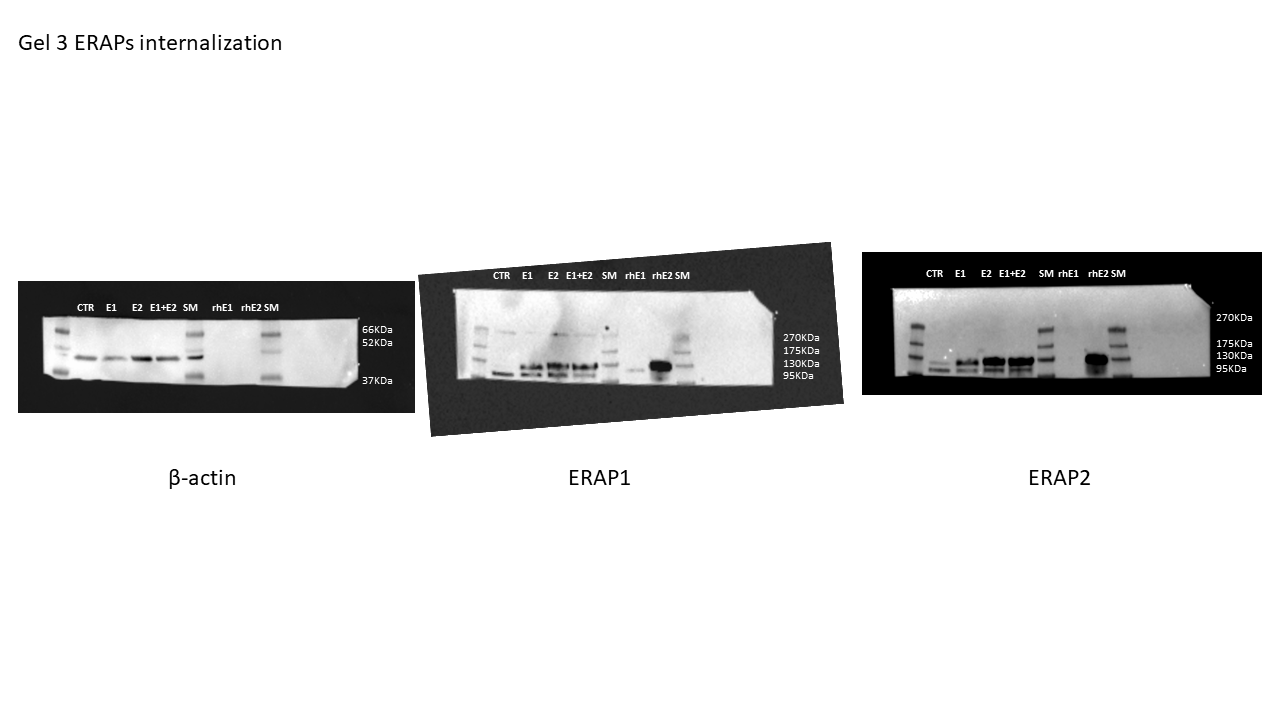

Supplement: Supplementary file 3 [file DataSheet1.zip › immagini/gel 3 ERAP internalization.tif]

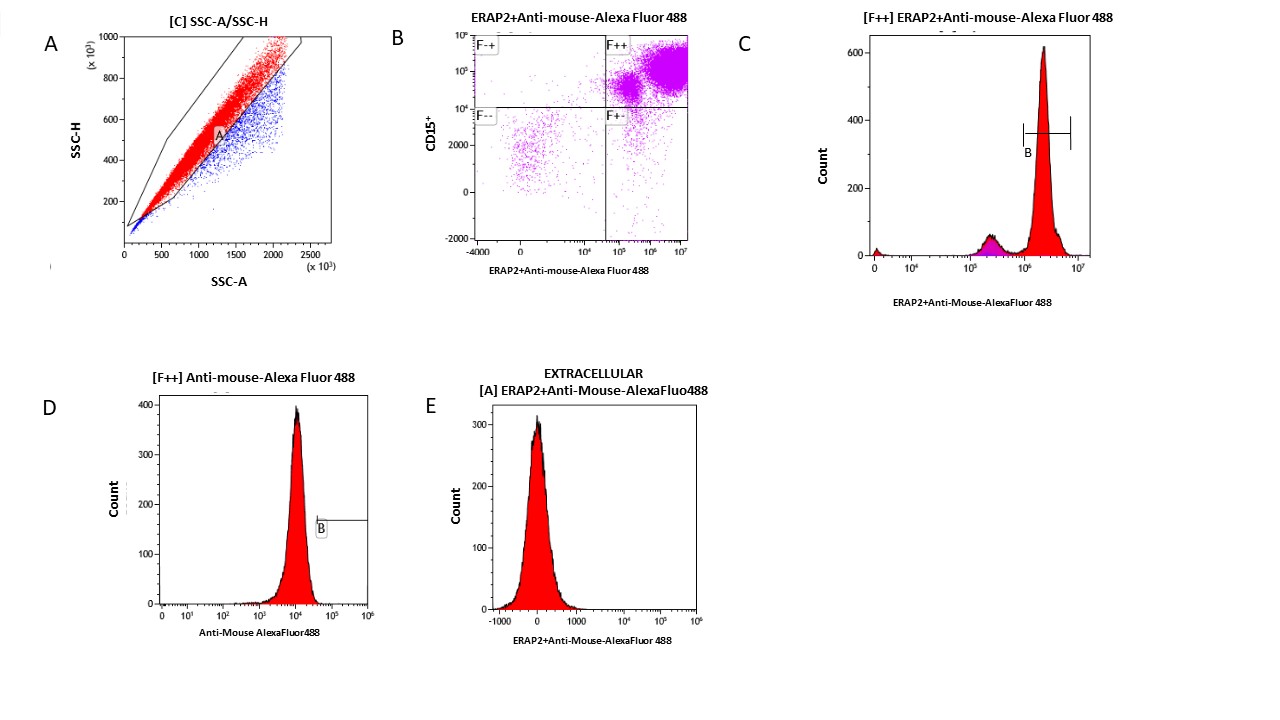

Supplement: Supplementary file 4 [file Image2.jpeg]
